# Dynamic properties of independent chromatin domains measured by correlation spectroscopy in living cells

**DOI:** 10.1186/s13072-016-0093-1

**Published:** 2016-12-24

**Authors:** Malte Wachsmuth, Tobias A. Knoch, Karsten Rippe

**Affiliations:** 1 Cell Biology and Biophysics Unit, European Molecular Biology Laboratory (EMBL), Meyerhofstrasse 1, 69117 Heidelberg, Germany; 2 Biophysical Genomics Group, Department of Cell Biology and Genetics, Erasmus Medical Center, Dr. Molewaterplein 50, 3015 GE Rotterdam, The Netherlands; 3 Research Group Genome Organization and Function, Deutsches Krebsforschungszentrum (DKFZ) & BioQuant, Im Neuenheimer Feld 280, 69120 Heidelberg, Germany

**Keywords:** Chromatin structure, Polymer model, Chromatin conformation capture carbon copy (5C), Targeted chromatin capture (T2C), Fluorescence correlation spectroscopy (FCS), Quantitative microscopy

## Abstract

**Background:**

Genome organization into subchromosomal topologically associating domains (TADs) is linked to cell-type-specific gene expression programs. However, dynamic properties of such domains remain elusive, and it is unclear how domain plasticity modulates genomic accessibility for soluble factors.

**Results:**

Here, we combine and compare a high-resolution topology analysis of interacting chromatin loci with fluorescence correlation spectroscopy measurements of domain dynamics in single living cells. We identify topologically and dynamically independent chromatin domains of ~1 Mb in size that are best described by a loop-cluster polymer model. Hydrodynamic relaxation times and gyration radii of domains are larger for open (161 ± 15 ms, 297 ± 9 nm) than for dense chromatin (88 ± 7 ms, 243 ± 6 nm) and increase globally upon chromatin hyperacetylation or ATP depletion.

**Conclusions:**

Based on the domain structure and dynamics measurements, we propose a loop-cluster model for chromatin domains. It suggests that the regulation of chromatin accessibility for soluble factors displays a significantly stronger dependence on factor concentration than search processes within a static network.

**Electronic supplementary material:**

The online version of this article (doi:10.1186/s13072-016-0093-1) contains supplementary material, which is available to authorized users.

## Background

The three-dimensional organization of chromosomes of eukaryotic interphase cells is emerging as an important parameter for the regulation of genomic function [1–4]. Beyond the mere storage of genetic information, the spatial structure fosters its compaction, replication and transcription on all scales ranging from the single base pair (bp) to ~100 Mbp of a whole chromosome. Chromatin interaction maps obtained by the chromatin conformation capture (3C) assay [5, 6] and derived methods like 5C, Hi-C [7] or T2C [8] provide detailed genome-wide information on the three-dimensional organization of the mammalian genome for cell ensembles [9–12] or even single cells [13]. These analyses suggest that the genome is organized into distinct topologically associating domains (TADs) [3, 11, 14]. They partition the genome into repressive and active chromatin regions, also referred to as subchromosomal domains [15, 16] and as concluded from a number of microscopy studies on the topology of active gene clusters [17–19] or the timing differences between early- and late-replicating DNA loci [20]. Notably, the spatial segregation of the genome into chromatin regions with different gene expression status is not simply the result of transcriptional activity. Rather, spatial chromatin organization actively participates in shaping cellular functions [4, 21–24]. Yet, details of the folding of the nucleosome chain into subchromosomal domains or TADs and entire chromosomes remain largely elusive. For the chromatin fiber, a variety of models covering a broad range from unordered and less compact to regular and more compacted states have been suggested [25–27], and likewise, for the higher-order folding of the fiber there is experimental evidence for both more ordered loop- or rosette-like [12, 28–31] and less ordered, e.g., fractal globule-like topologies [10].

Despite the impressive advancements in the field, details on the organization and dynamic properties of chromatin in single living cells are elusive. However, the plasticity of chromatin organization is a central determinant of genome function as it modulates access of factors to the genome and targets them to biologically active subcompartments [32]. In addition to large-scale chromosomal movements [33], local chromatin dynamics are mostly studied by tracking of few genomic loci and chromatin-associated or chromatin-embedded molecules and particles as reviewed previously [34–37]. The resulting translocation data can be quantified as mean-squared displacement (MSD) versus time curves to extract apparent velocities or diffusion coefficients. These studies revealed spatially confined movements of tagged chromatin loci as intuitively evident for a segment of a polymer without center-of-mass translocation [38–40]. However, extending this approach to a systematic analysis of endogenous chromatin loci faces a number of limitations. Imaging-based techniques typically require the labeling of specific genomic regions using repetitive, e.g., *lac*O operator arrays integrated into the genome at random or defined positions [41]. These arrays are big compared to the dimensions of the structures under investigation and potentially alter their architecture. Furthermore, this approach is limited in its time resolution to the image acquisition time, which is typically in the range of 50 ms or higher. At the molecular level, methods like fluorescence recovery after photobleaching (FRAP), continuous photobleaching (CP) and fluorescence correlation spectroscopy (FCS) provide information on the binding of proteins to chromatin and on their mobility within the chromosomal environment on the microsecond to minute time scale [42, 43]. However, with these methods no information on the dynamics of nucleosome chains and higher-order domains has yet been obtained. While biophysical polymer models have been widely used to quantitatively describe and directly or inversely compare 3D chromatin structure to experimental data as reviewed recently [44, 45], they mostly do not include dynamics. Thus, our current knowledge is lacking both experimental information and theoretical treatment of the conformational dynamics of chromatin in vivo that is important for the understanding of the differential readout of DNA sequence information or interactions between different genomic loci.

In a number of studies, intramolecular dynamics have been investigated by FCS [46, 47]. By uncoupling the center-of-mass diffusion from higher-order relaxation modes via trapping or tracking [48, 49], a series representation of relaxation modes was obtained to describe the internal dynamics of double-stranded DNA in vitro [49–51]. In this manner, the MSD of polymer segments can be described as confined diffusion relative to the center of mass. When taking into account hydrodynamic interactions, molecules like long DNA chains with a sufficiently large ratio of contour to persistence length, i.e., ‘soft’ polymers, show Zimm relaxation behavior [52].

Here, we combine for the first time the topological interpretation of 3C-derived data from large ensembles of fixed cells with the measurement of mesoscale chromatin dynamics in individual living cells. We confirm the formation of loop clusters in TADs from contact probability maps (5C, T2C) from other studies ([11, 53], NCBI GEO accession GSE35721) pointing to rosettes as a prominent structural feature of such topologically independent domains. By applying FCS, we measured chromatin dynamics extracted from fluorescence intensity fluctuations by exploiting the linker histone variant H1.0 tagged with EGFP (H1-EGFP) as a proxy for chromatin movement. H1 is particularly suited for this purpose since it decorates chromatin globally and reflects its density but binds only transiently [54, 55] such that photobleached molecules are constantly replaced by fluorescent ones. We found distinct chromatin relaxation times, hallmarking the presence of dynamically and topologically independent chromatin units with an average genomic content of ~1 Mb. Treatment of cells with trichostatin A (TSA) and azide-induced ATP depletion resulted in decelerated relaxations, revealing chromatin decondensation and compaction, respectively, hence delivering insight into factors that change chromatin dynamics. Based on the experimental data, an analytical polymer model was developed. It correctly describes both the contact probability maps from 3C-based ensemble analysis and the internal dynamics of chromatin domains observed by FCS. We hypothesize that these domains might be TADs. From the dynamic properties measured, we infer that the different time scales of structural reorganization and particle dynamics provide an additional regulatory layer for targeting soluble nuclear factors to chromatin subcompartments.

## Results

### A loop-cluster substructure domain model shows good agreement with experimental 5C and T2C data

**Fig. 1:**
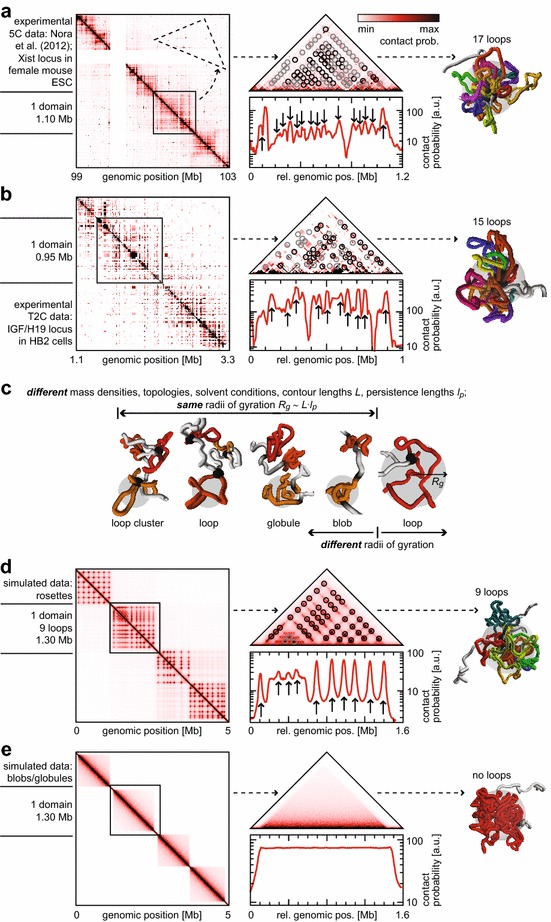
5C and T2C analysis and polymer modeling. **a** Genomic contact probability matrix for experimental 5C data [11]. The *black square* highlights a domain that is further studied. The *dashed profile* shows how the non-redundant triangular representation was extracted. We could identify loop bases (*circles*) with higher (*black*) or smaller (*gray*) significance. The 1D plot represents the global projection of the highlighted domain. *Arrows* indicate identified loop bases. The extracted loops allowed to simulate and visualize an exemplary configuration and to compute the *R*_*g*_. **b** Same as **a**, but for experimental T2C data [53]. **c** The different chromatin domain conformations probed in this study to model the FCS data: blob, globule, loop and loop cluster. The radius of gyration *R*_*g*_(*gray circle*) of domains depends on physical parameters, solvent conditions and the topology of the underlying chromatin fiber. It determines the characteristic time constants of internal relaxation kinetics observed in this study. **d** Same as **a**, but for a model configuration of the loop-cluster conformation under theta-solvent conditions (see Additional file 1: Supplementary Text). **e** Same as **a**, but for a model configuration of the globular conformation

To gain insight into the topological organization of chromatin, we applied a simple domain and peak detection approach to 5C data of a 4.5-Mb region containing the Xist gene crucial for X inactivation in female mouse embryonic stem cells [11] and T2C data of a 2.2-Mb region of the IGF/H19 locus in human HB2 cells [8]. Figure 1a shows the analysis of the experimental 5C data set for which we confirmed the existence of TAD-like domains such as the highlighted ~1.1-Mb region that emerged as square-shaped regions of increased internal contact probability as expected [7, 11, 14, 56]. A one-dimensional projection over the whole domain region yielded primary peaks corresponding to genomic sites involved in loop formation (Additional file 1: Fig. S1). Orthogonal local projections around each so-determined peak revealed all partner sites with which it interacts to form loops. We obtained 17 primary peaks within this domain (Additional file 1: Fig. S2). Most of them also emerged in the local projections, strongly indicating that this domain consisted to a significant extent of an intricately tied loop cluster such as a rosette. We followed the same procedure for an experimental T2C data set from Knoch et al. [53] (Fig. 1b). Again, we found domains such as the highlighted ~0.95-Mb region and 15 primary peaks within this domain (Additional file 1: Fig. S3), most of which also emerged in the local projections, again indicating a rosette-like loop-cluster organization of the domain.

### Domain configurations are well described with a quantitative polymer model

While these examples support the notion of loop-induced domain formation, also less ordered crumpled, globular or ordinary domain structures were suggested previously [10, 12, 44]. Accordingly, we derived a quantitative polymer model that describes 4 different domain topologies to comprehensively cover the previously proposed features of chromatin domain organization (Fig. 1c; Additional file 1: Fig. S4): Scaling laws from polymer theory [57] suggest that chromatin adopts the shape of a chain of topologically and dynamically independent domains under the semi-dilute conditions met in mammalian interphase nuclei (see Additional file 1: Supplementary Text for more details). Thus, we first assumed the formation of such blobs, i.e., globular subchains of the full chromosome that are significantly shorter and behave like independent, almost self-penetrating molecules (so-called theta-solvent conditions where repulsive and attractive segment–segment interactions compensate each other), connected with a linker. Second, the formation of space-filling fractal or crumpled globules [10, 44] was evaluated. Third, we assumed the formation of single or rosette-like branched loops [29, 30, 58, 59] under theta-solvent conditions. Fourth, the same topology was used, but under so-called good-solvent conditions where the excluded volume interaction between segments dominates and the structure appears swollen as compared to theta-solvent conditions. The physical contour length *L* of the chromatin fiber contained in the domain is directly related to DNA content and density, and the persistence length *l*_*p*_ is a measure for the fiber flexibility. Together with the number of contained loops *f*, these parameters determine the radius of gyration *R*_*g*_, which characterizes the volume effectively occupied by the domain—Additional file 1: Eq. S14, S20, S22, S24—according to Eq. 1:

1 $$R_{g}^{2} = \left\{ {\begin{array}{*{20}l} {\frac{{L \cdot l_{p} }}{6}\left( {\frac{2f - 1}{{f^{2} }}} \right)} \hfill & {\text{loop-rosette conformation, theta-solvent conditions,}} \hfill \\ {\frac{{L^{{{6 \mathord{\left/ {\vphantom {6 5}} \right. \kern-0pt} 5}}} \cdot l_{p}^{{{4 \mathord{\left/ {\vphantom {4 5}} \right. \kern-0pt} 5}}} }}{9.59}\left( {\frac{1.92f - 0.92}{{f^{{{{11} \mathord{\left/ {\vphantom {{11} 5}} \right. \kern-0pt} 5}}} }}} \right)} \hfill & {\text{loop-rosette conformation, good-solvent conditions,}} \hfill \\ {\frac{{L^{{{2 \mathord{\left/ {\vphantom {2 3}} \right. \kern-0pt} 3}}} \cdot l_{p}^{{{4 \mathord{\left/ {\vphantom {4 3}} \right. \kern-0pt} 3}}} }}{1.76}} \hfill & {\text{globular conformation,}} \hfill \\ {\frac{{L \cdot l_{p} }}{3}} \hfill & {{\text{blob conformation}} .} \hfill \\ \end{array} } \right.$$

An estimation of stochastic contact probabilities—Additional file 1: Eq. S25—directly allowed to compute 5C-/T2C-like contact probability maps. Figure 1d, e shows such maps for both the theta-solvent loop-cluster and the globular conformation (Additional file 1: Supplementary Text), i.e., for a 5-Mb stretch comprising 4 rosette-like loop clusters and 4 globular domains, respectively, linked with a relaxed chromatin stretch. Here too, domains emerged as square-shaped regions of increased internal contact probability. The highlighted rosette domain in Fig. 1d was computed assuming 10 loops (three with positional noise). Applying the same analysis as above allowed us to quantitatively retrieve the topological details used for the simulation: Some ties were found in both projection directions, others, especially those with positional noise, less reliably in only one direction. Using the topology retrieved, we performed Monte Carlo (MC) simulations of the domain (with one example visualized, Fig. 1d) to yield its radius of gyration of ~240 nm. The globular domain model yielded a smaller radius of gyration of 210 nm but was incompatible with the experimental data since no peaks were detected (Fig. 1e). To further validate the analysis and simulation pipeline, we used the topology obtained from the experimental 5C and T2C data to re-calculate the experimental contact probability maps, which were in good agreement with the initial ones (Additional file 1: Figs. 5, 6). From MC simulations, we found a radius of gyration of ~240 nm for the domain highlighted in the 5C data set and of ~220 nm in the T2C data set (Fig. 1a, b). In summary, a much better agreement with the experimental data was found for the loop-cluster model than for the globular domain model.

### Chromatin fiber dynamics can be evaluated with FCS of transiently bound linker histone

**Fig. 2:**
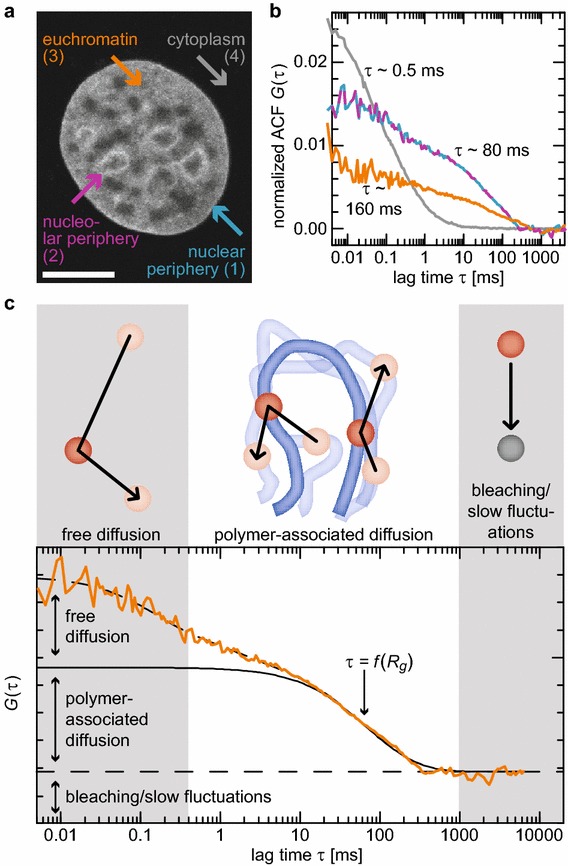
Observation and interpretation of chromatin dynamics seen with FCS. **a** MCF7 cell stably expressing H1-EGFP with typical localizations for FCS measurements used throughout this study. **b** Typical ACFs obtained at the different locations, showing fast decay due to free diffusion in the cytoplasm and slower decay in the nuclear and nucleolar periphery and even slower decay in euchromatin. **c** Different regimes of the ACFs correspond to different processes: A fast initial decay results from free H1.0 diffusion, followed by a slow decay due to chromatin-associated diffusion or relaxation, whose time constant depends on *R*_*g*_. Slower processes such as photobleaching do not show up

5C and T2C analyses yield structural information from large ensembles of fixed cells. However, the dynamic properties of the observed domains remain elusive. Therefore, we measured chromatin dynamics with FCS using the approach depicted in Fig. 2. The dynamics of linker histone H1-EGFP were determined in the cytoplasm, in less chromatin-dense areas in the nucleus referred to as ‘euchromatin’ and in denser chromatin regions in the nuclear and in the nucleolar periphery referred to as ‘heterochromatin’ in the following [60] (Fig. 2a; Additional file 1: Supplementary Text, Fig. S7 for details on classification). In the cytoplasm, we obtained a fast decay with a characteristic diffusion coefficient of *D* ≈ 20 µm^2^ s^−1^ that we assigned to free diffusion of H1.0 (Fig. 2b). Inside the nucleus, the autocorrelation functions (ACFs) decayed bimodally. The first component decayed within 1 ms owing to a freely diffusive fraction. The second, slower decaying contribution was about two magnitudes slower between ~90 and ~160 ms depending on the previously defined nuclear subcompartments used for the measurement. We assigned these slower decays to chromatin-associated movements (Fig. 2c): Distinct relaxation times of chromatin measured by FCS clearly indicated the existence of topologically and dynamically independent chromatin units of a certain scale. The detailed analysis of H1.0 chromatin interactions with FRAP and FCS experiments as well as FCS measurements of H2A and H2B core histones (see below) further corroborated this. Processes that occur at times above 1 s like photobleaching or cellular movements were not detected in FCS due to the short effective measurement time (Additional file 1: Supplementary Text, Fig. S8). Thus, combining FCS measurements with hydrodynamic polymer models should enable us to extract the size of these domains as well as their topologies and physical properties (Fig. 1c).

### Both transient chromatin-binding modes of H1.0 are slower than fluctuations seen by FCS

**Fig. 3:**
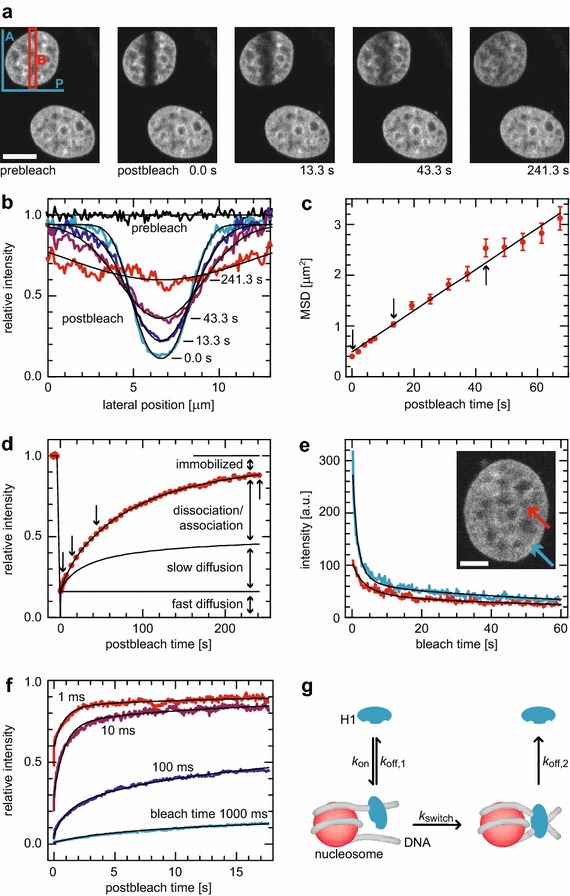
Photobleaching analysis of H1.0-chromatin binding. **a** Imaging FRAP experiment of H1-EGFP expressed in an MCF7 cell. Strip B (*red*) is bleached into the nucleus. The redistribution is followed over time and analyzed in different ways. **b** Averaging along the direction of the long strip dimension A (*blue* in **a**), plotting the profile perpendicularly in direction P and normalizing to the prebleach distribution (Additional file 1: Fig. S11) provided time-dependent profiles. They were fitted with Additional file 1: Eq. S91 to yield the MSD over time. **c** From a linear fit, apparent diffusion coefficients around 10^−3^ µm^2^ s^−1^ were extracted. **d** However, the apparent diffusion model, already comprising a fast reaction–diffusion scheme, did not explain exhaustively the intensity time trace obtained by averaging over the bleach region B in **a**. It required additional fast diffusive, transiently binding and immobilized fractions of the molecules for comprehensive modeling of the recovery data. However, a closed expression for a full reaction–diffusion scheme with two immobilization states cannot be derived. **e** We used continuous fluorescence photobleaching (CP), for which a closed expression with two bound states existed and which also allowed to address more specifically the localization types used in this study. This yielded a short-lived (residence time ~1 s) and a long-lived (~2 min) type of immobilization, whose fractions and detailed properties depended on localization and treatment of the cells with ATP or azide. **f** Globally fitting point FRAP experiments featuring bleach times series confirmed the CP and imaging FRAP results. **g** Resulting model of H1.0 binding: molecules bind to the DNA entry–exit sites of nucleosomes with rate *k*_on_. Either they rapidly dissociate again with rate *k*_off,1_, or they engage with rate *k*_switch_ to the longer-lived conformation, from which they dissociate eventually with rate *k*_off,2_

To further rule out that the relaxations in FCS were association–dissociation events, we precisely quantified transient chromatin binding of H1.0 labeled with EGFP with fluorescence recovery after photobleaching (FRAP) experiments. We bleached a strip through the cell nucleus (Fig. 3a) in non-, TSA- and azide-treated cells. The mobility of H1-EGFP was analyzed by fitting the bleach profile (Fig. 3b; Additional file 1: Fig. S11) with Additional file 1: Eq. S91 to follow its broadening as given by its width *σ*. From linear regressions of *σ* ^2^ plotted versus time, apparent diffusion coefficients of *D*_app_ = (10 ± 5)·10^−3^ µm^2^ s^−1^, (12 ± 4)·10^−3^ µm^2^ s^−1^ and (10 ± 3)·10^−3^ µm^2^ s^−1^ were derived (non-, TSA- and azide-treated; Fig. 3c). These values were at least two orders of magnitude smaller than those for free H1-EGFP (*D* ≈ 20 µm^2^ s^−1^) and at least one order of magnitude larger than the apparent diffusion coefficient of chromatin loci obtained by tracking [35]. Thus, the apparent diffusion process represents coupled diffusion and binding as reported previously [61]. Inspecting the integrated fluorescence intensity in the bleached region over time revealed that the expected intensity change calculated for diffusive redistribution using these *D*_app_ values differed significantly from the experimentally observed behavior (Fig. 3d; Additional file 1: Fig. S12). Therefore, at least two different binding states must be present, with *D*_app_ comprising the kinetics of the faster one. Accordingly, the intensity change was fitted with the uncoupled diffusion and binding model given in Additional file 1: Eq. S92. It includes fast free diffusion for which recovery is already complete at the first postbleach time point. The second term covers fast binding and diffusion, while slow dissociation was taken into account separately [62, 63]. This yielded free diffusive fractions of 6 ± 3, 11 ± 4 and 18 ± 12 % and slow dissociation rates of (8.8 ± 2.6)·10^−3^ s^−1^, (13.7 ± 6.3)·10^−3^ s^−1^ and (12.2 ± 2.3)·10^−3^ s^−1^ for non-, TSA-, and azide-treated cells, respectively.

As an independent confirmation of the above results and to extract also the faster dissociation rate, we conducted a continuous photobleaching (CP) analysis (Fig. 3e). The much higher spatial resolution of CP allowed to address local differences in H1-EGFP mobility. Fitting CP curves with Additional file 1: Eq. S93 confirmed the existence of two chromatin-binding states. The analysis yielded fast dissociation rates of 1.05 ± 0.13 s^−1^ in heterochromatin and 0.76 ± 0.21 s^−1^ in euchromatin of non-treated cells and fractions of 18 ± 2 and 31 ± 9 %, respectively, of the molecules in this association state. Point FRAP (Fig. 3f) confirmed these results by performing series of experiments acquired at single spots in euchromatin with different lengths of the bleach segment [43]. The resulting dissociation rates of (8.2 ± 3.5)·10^−3^ s^−1^ and 0.83 ± 0.20 s^−1^ for the two binding states were in good agreement with the above findings.

Using this and the previously reported presence of two DNA binding domains in H1 [64], we suggest the following model (Fig. 3g): One binding domain of H1.0 interacts with the entry–exit site of DNA at the nucleosome and either dissociates quickly or engages the second domain to form a longer-lived binding state, from which it dissociates again later. Deriving the rate equations for the different binding states allowed us to calculate the remaining parameters in differently treated cells [65] and in euchromatin and heterochromatin (Additional file 1: Eq. S95; Table 1): The residence time of H1.0 in the short-lived binding state was ~1 s, whereas the average residence time on chromatin was ~4 s. Thus, the fluctuations observed with FCS with relaxation times of ~100 ms did not result from association/dissociation events but rather from chromatin dynamics. Despite our purely intensity-based distinction of euchromatin and heterochromatin, we found a higher effective affinity of H1.0 to heterochromatin as expected [66].

**Table 1:** Properties of histone H1.0 binding to chromatin obtained with FRAP and CP

	*f*_free_ [%]	*f*_short_ [%]	*f*_long_ [%]	*k*_on_ (s^−1^)	*k*_off,1_ (s^−1^)	*k*_switch_ [10^−3^s^−1^]	*k*_off,2_ [10^−3^s^−1^]
*Untreated*							
Heterochromatin	6 ± 3	18 ± 2	76 ± 4	3.3 ± 1.7	1.05 ± 0.13	33 ± 12	8 ± 3
Euchromatin	6 ± 3	31 ± 9	63 ± 9	4.0 ± 2.5	0.76 ± 0.21	16 ± 8	8 ± 3
TSA-treated	11 ± 4	89 ± 4		2.1 ± 1.2	0.89 ± 0.22	31 ± 11	12 ± 4
ATP-depleted	18 ± 12	82 ± 12		1.3 ± 1.0	0.89 ± 0.22	27 ± 13	12 ± 2

(mean value ± standard deviation)

*f*_free_—free fraction, *f*_short_—shortly bound fraction, *f*_long_—long-bound fraction, *k*_on_—association rate, *k*_off_—dissociation rate, *k*_switch_—switching rate, TSA—Trichostatin A, ATP—adenosine triphosphate

### FCS measurements of core histones H2A and H2B confirm chromatin fluctuations with ~100 ms relaxation times

**Fig. 4:**
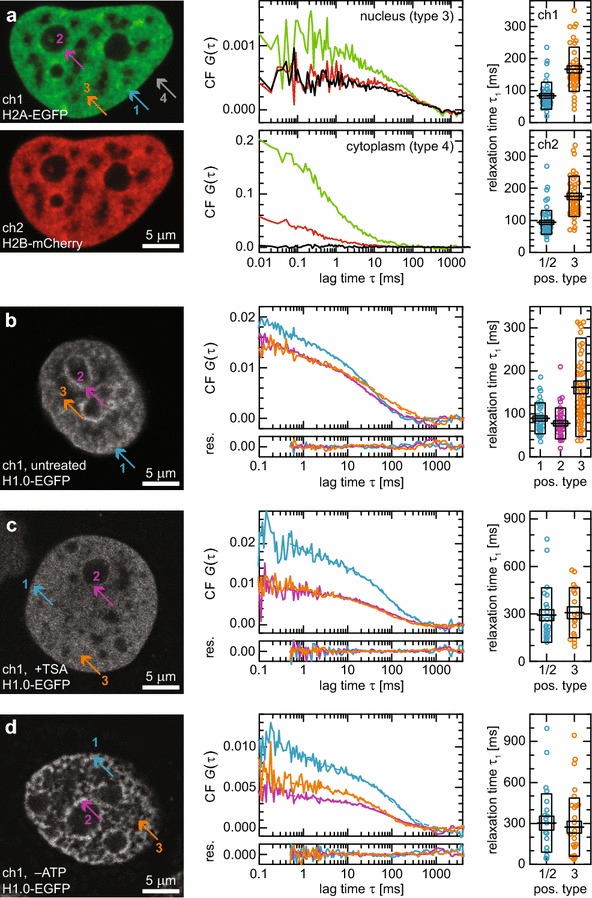
FCS analysis of chromatin dynamics. **a** HeLa cell expressing H2A–EGFP (transient) and H2B–mCherry (stable). The correlation plots show H2A–EGFP ACFs (*green*), H2B–mCherry ACFs (*red*) and their CCF (*black*) acquired in the nucleus (euchromatin—3) and in the cytoplasm (4), revealing significant cross-correlation in the nucleus, but not in the cytoplasm. Fitting them with a relaxation model for loop-rosette-structured polymers under theta-solvent conditions yielded a significant difference in relaxation time distribution between hetero-(1/2) and euchromatin (3) both for H2A (ch1) and H2B (ch2). **b** Untreated MCF7 cell expressing H1-EGFP. At the three positions (nuclear periphery—1, *blue*; nucleolar periphery—2, *purple*; euchromatin—3, *orange*), the corresponding ACFs were acquired. Fitting them like in **a** (res—residuals) yielded a significant difference in relaxation time distribution between hetero- (1, 2) and euchromatin (3). **c** Same as **b**, but cells were treated with TSA, resulting in globally increased relaxation times without significant differences between 1, 2 and 3. **d** Same as **b**, but cells were ATP-depleted, resulting in globally increased relaxation times without significant differences between 1, 2 and 3

To confirm that the ~100 ms relaxation times indeed represent chain dynamics and not unbinding events or photophysical effects of the fluorescent protein domains, we repeated the measurements in HeLa cells stably expressing histone H2B–mCherry fusions and transiently expressing H2A–EGFP fusions at a ratio of ~5 % to the corresponding endogenous protein [60]. As expected, both the spatial chromatin distribution and the relaxation times were virtually the same for both histones (Fig. 4a). The measured values for nuclear relaxation times were in excellent agreement with H1.0 measurements, which are elucidated in detail in the following section. Fitting the ACFs with model functions for chromatin relaxation based on the comprehensive set of 4 polymer models (Eq. 3) allowed us to quantify the differences between the intranuclear positions studied: In heterochromatin, we obtained 83±7 and 94±6 ms for H2A–EGFP and H2B–mCherry, respectively, as first-order mode relaxation time under theta-solvent conditions (see next section for details and Table 2 for good-solvent and globular conditions). Corresponding values in euchromatin were approximately twofold slower with 165±11 and 174±10 ms, respectively, in contrast to the expectation that in lower density regions, relaxations would be faster. Importantly, the fluctuations showed a pronounced cross-correlation due to the co-diffusion of H2A and H2B simultaneously integrated into nucleosomes and chromatin. In contrast, there was no cross-correlation in the cytoplasm as expected. These observations corroborate our conclusion that chromatin dynamics are the source of the observed fluctuations. It can be ruled out that they are due to blinking of fluorescent protein domains because this would not result in a cross-correlated signal. Furthermore, the cross-correlation cannot result from spectral cross-talk because this would yield high cross-correlation in the cytoplasm, too.

**Table 2:** Dynamic and structural parameters of histone-FP-labeled chromatin domains obtained with FCS at different nuclear localizations

		*n*	Loop-rosette, blob; theta-solvent conditions^a^			Loop-rosette; good-solvent conditions^b^			Globular^b^	
			*τ*_1_ (ms)	*R*_*g*_ (nm)	*gc* (Mb)	*τ*_1_ (ms)	*R*_*g*_ (nm)	*gc* (Mb)	*R*_*g*_ (nm)	*gc* (Mb)
H1-EGFP untreated	Perinuclear	35	91 ± 6	245 ± 5	0.80–1.12	100 ± 6	289 ± 6	1.31–1.83	240 ± 5	0.75–1.05
	Perinucleolar	34	78 ± 6	234 ± 6	0.70–0.98	94 ± 5	283 ± 5	1.23–1.73	235 ± 4	0.71–0.99
	Euchromatin	62	161 ± 15	297 ± 9	0.83–1.16	191 ± 20	359 ± 12	1.47–2.05	298 ± 10	0.84–1.17
H2A-EGFP untreated	Perinucle(ol)ar	84	83 ± 7	238 ± 4	0.73–1.03	90 ± 8	279 ± 4	1.18–1.65	232 ± 4	0.68–0.95
	Euchromatin	84	165 ± 11	299 ± 9	0.85–1.19	188 ± 14	356 ± 13	1.43–2.00	297 ± 11	0.83–1.16
H2B-mCherry untreated	Perinucle(ol)ar	84	94 ± 6	249 ± 4	0.84–1.18	102 ± 7	291 ± 4	1.34–1.88	242 ± 3	0.77–1.08
	Euchromatin	84	174 ± 10	304 ± 9	0.89–1.25	195 ± 12	361 ± 12	1.49–2.09	300 ± 10	0.86–1.12
H1-EGFP TSA-treated	Perinucle(ol)ar	25	292 ± 34	362 ± 14	1.65–2.31	366 ± 49	445 ± 20	3.07–4.30	370 ± 17	1.77–2.47
	Nucleoplasm	18	307 ± 37	368 ± 15	1.74–2.43	384 ± 51	453 ± 20	3.24–4.54	377 ± 17	1.87–2.61
H1-EGFP ATP-depleted	Perinucle(ol)ar	17	303 ± 51	367 ± 21	1.72–2.41	388 ± 74	454 ± 29	3.26–4.57	378 ± 24	1.88–2.64
	Nucleoplasm	25	278 ± 43	356 ± 18	1.57–2.20	351 ± 59	439 ± 25	2.95–4.13	365 ± 21	1.69–2.37

(mean value ± standard error; min. value–max. value)

*τ*_1_—decay time of the first polymer relaxation mode, *R*_*g*_—radius of gyration of topologically and dynamically independent chromatin domain, *gc*—genomic content of topologically and dynamically independent chromatin domain

^a^Relaxation times and radii of gyration are numerically identical for loop-rosette conformation under theta-solvent conditions and for blob conformation

^b^Relaxation times are numerically identical for loop-rosette conformation under good-solvent conditions and for globular conformation

### Polymer relaxation modes seen by autocorrelation analysis reflect persistence length, mass density and topology of chromatin domains

To decompose the autocorrelation analysis into parameters that describe features of polymer domains, the Rouse–Zimm model was applied for a quantitative characterization of domain dynamics [52]. Independent relaxation modes represent distinct characteristic times *τ*_*p*_ and amplitudes $a_{p} = \langle {{\mathbf{X}}_{p}^{2} } \rangle$ that are observable in the FCS experiments. These parameters depend on topology, solvent conditions, viscosity *η*_s_, temperature *T*, Boltzmann constant *k*_*B*_ and radius of gyration *R*_*g*_ (see Additional file 1: Supplementary Text for more details):

2 $$\begin{array}{*{20}l} \begin{aligned} \tau_{1} \approx 6.111\frac{{\eta_{s} R_{g}^{3} }}{{k_{B} T}} ,\quad \tau_{p} = \frac{{\tau_{1} }}{{p^{{{3 \mathord{\left/ {\vphantom {3 2}} \right. \kern-0pt} 2}}} }},\quad a_{p} \approx 0.152\frac{{R_{g}^{2} }}{{p^{2} }}, \hfill \\ \hfill \\ \end{aligned} \hfill & \begin{aligned} {\text{loop-rosette conformation,}} \hfill \\ {\text{theta-solvent conditions,}} \hfill \\ \hfill \\ \end{aligned} \hfill \\ \begin{aligned} \tau_{1} \approx 4.114\frac{{\eta_{s} R_{g}^{3} }}{{k_{B} T}} ,\quad \tau_{p} = \frac{{\tau_{1} }}{{p^{{{{17} \mathord{\left/ {\vphantom {{17} {20}}} \right. \kern-0pt} {20}}}} }},\quad a_{p} \approx 0.172\frac{{R_{g}^{2} }}{{p^{{{9 \mathord{\left/ {\vphantom {9 4}} \right. \kern-0pt} 4}}} }}, \hfill \\ \hfill \\ \end{aligned} \hfill & \begin{aligned} {\text{loop-rosette conformation,}} \hfill \\ {\text{good-solvent conditions,}} \hfill \\ \hfill \\ \end{aligned} \hfill \\ \begin{aligned} \tau_{1} \approx 7.151\frac{{\eta_{s} R_{g}^{3} }}{{k_{B} T}} ,\quad \tau_{p} = \frac{{\tau_{1} }}{p} ,\quad a_{p} \approx 0.236\frac{{R_{g}^{2} }}{{p^{{{5 \mathord{\left/ {\vphantom {5 3}} \right. \kern-0pt} 3}}} }}, \hfill \\ \hfill \\ \end{aligned} \hfill & \begin{aligned} {\text{globular conformation,}} \hfill \\ \hfill \\ \end{aligned} \hfill \\ \begin{aligned} \tau_{1} \approx 5.849\frac{{\eta_{s} R_{g}^{3} }}{{k_{B} T}} ,\quad \tau_{p} = \frac{{\tau_{1} }}{{p^{{{3 \mathord{\left/ {\vphantom {3 2}} \right. \kern-0pt} 2}}} }},\quad a_{p} \approx 0.152\frac{{R_{g}^{2}} }{{p^{2} }}, \hfill \\ \hfill \\ \end{aligned} \hfill & \begin{aligned} {\text{blob/linear conformation;}} \hfill \hfill \\ {\text{mode number }}p = 1,2,3, \ldots \hfill \\ \hfill \\ \end{aligned} \hfill \\ \end{array}$$

These relaxations result in local concentration fluctuations of segments even when the center-of-mass translocation is negligible. An obvious way to study such fluctuations is their evaluation by autocorrelation analysis as conducted for FCS measurements. Relaxation modes are independent of each other and have exponentially decaying position correlation functions [52]. Thus, each mode is represented by a diffusion process in a harmonic potential, which is an Ornstein–Uhlenbeck process, the simplest example of a stationary Markovian process with Gaussian probability distribution at all times [67]. To this theoretical framework, the FCS formalism was applied [68, 69] (Additional file 1: Supplementary Text), yielding the autocorrelation function

3 $$ G\left( \tau \right) \propto a_{p} \left[ {\left( {1 + \frac{{1 - \exp \left[ { - {\tau \mathord{\left/ {\vphantom {\tau {\tau_{p} }}} \right. \kern-0pt} {\tau_{p} }}} \right]}}{\upsilon }} \right)^{ - 1} \left( {1 + \frac{{1 - \exp \left[ { - {\tau \mathord{\left/ {\vphantom {\tau {\tau_{p} }}} \right. \kern-0pt} {\tau_{p} }}} \right]}}{{\kappa^{2} \upsilon }}} \right)^{{ - {1 \mathord{\left/ {\vphantom {1 2}} \right. \kern-0pt} 2}}} } \right.\left. { - \left( {1 + \frac{1}{\upsilon }} \right)^{ - 1} \left( {1 + \frac{1}{{\kappa^{2} \upsilon }}} \right)^{{ - {1 \mathord{\left/ {\vphantom {1 2}} \right. \kern-0pt} 2}}} } \right]. $$

Here, $\upsilon = {{\tau_{D} } \mathord{\left/ {\vphantom {{\tau_{D} } {\tau_{p} }}} \right. \kern-0pt} {\tau_{p} }}$ is the ratio of diffusion correlation and relaxation time and $\kappa = {{z_{0} } \mathord{\left/ {\vphantom {{z_{0} } {w_{0} }}} \right. \kern-0pt} {w_{0} }}$ the structure parameter (Methods). Polymer relaxation was thus modeled by summing over $p = 1,2,3, \ldots$ of Eq. 3. The relaxation time *τ*_1_ from a fit of the model function to experimental data yielded the radii of gyration according to Eq. 2 with the nuclear solvent viscosity determined independently (Additional file 1: Supplementary Text). For known genomic content, a well-defined relationship between chromatin persistence length, mass density and domain topology such as the number of loops in a cluster/rosette can be established. Thus, the formalism links structural domain parameters from 3C-derived methods with dynamic features measured by FCS.

### FCS measurements of chromatin dynamics reveal different states of domain organization in hetero- and euchromatin

Fitting the ACFs with the polymer models (Eq. 1–3) allowed us to quantitatively determine chromatin relaxations times and other polymer parameters at different intranuclear positions and conditions (Fig. 4b; Table 2): In heterochromatin, e.g., at the nuclear or the nucleolar periphery, we obtained 90 ± 6 and 78 ± 6 ms, respectively, as first-order mode relaxation time under theta-solvent conditions. In the rest of the nucleus, in euchromatin, we measured 161 ± 15 ms, i.e., approximately twofold bigger values. Independent of the actual topological conformation, this can only be explained with a weaker local confinement of euchromatin due to a lesser degree of domain compaction because a purely chromatin density-driven relaxation would be faster in euchromatin compared to heterochromatin. In other words, comparing the relaxation with the oscillation of a bead on a string, the oscillation time is longer for a weaker string. Thus, the more open and less compact euchromatin can be compared to a weaker, more open string and the more compact heterochromatin to a stronger, more compact one.

After treatment of the cells with TSA, chromatin became hyperacetylated and adopted a decondensed state of the chromatin fiber [70, 71]. This process resulted in a homogeneous nuclear morphology and chromatin density distribution (Fig. 4c). The differences in chromatin relaxation at different nuclear loci vanished. The relaxations slowed down to time constants of 292 ± 34 ms at peripheral and 307 ± 37 ms at central nuclear positions (under theta-solvent conditions; Fig. 4c; see Table 2 for a summary of the different conformations). These values were even higher than those measured for euchromatin of untreated cells and indicated a further reduction in local confinement and an increased genomic content of domains.

The dynamics changed numerically similarly upon ATP depletion after treatment of the cells with azide. Here, however, the chromatin distribution became more aggregated with a less homogeneous morphology (Fig. 4d). The differences in chromatin relaxation vanished and the relaxations slowed down, resulting in time constants of 303 ± 51 ms in peripheral and 278 ± 43 ms in central positions (theta-solvent conditions, Fig. 4d; see Table 2 for a summary of the different conformations). This and the structural differences as seen in the images argue for increased sizes of domains due to agglutination effects. Interestingly, fundamentally different processes—decondensation and aggregation—result in the same effect of effective growth of independent domains. However, in the former case, the domains are distributed more and in the latter case less homogeneously than in untreated cells.

### FCS measurements of chromatin dynamics identify 1-Mb-sized dynamic domains

**Fig. 5:**
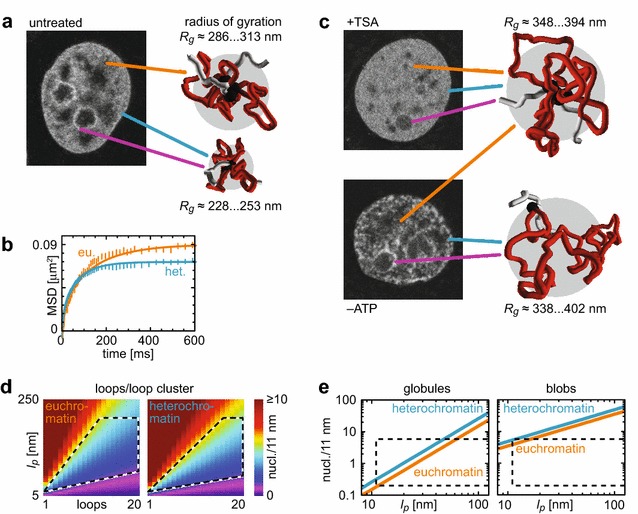
Physical properties and dynamics of domain structure. **a** Radii of gyration for the three different localization classes as extracted from the FCS data in Fig. 4 for untreated cells. The given range covers the results from the blob and the loop-cluster conformation under theta-solvent conditions and from the globular conformation and reveals differences in local domain size between euchromatin and heterochromatin. **b** MSD plots for typical chromatin segments in hetero- (het) and euchromatin (eu) calculated (*straight lines*) using the loop-rosette model under theta-solvent conditions and the radii of gyration from **a** and extracted from typical FCS measurements (*symbols*), showing confined diffusion on the 100 ms and 100 nm time and length scale. **c** Same as **a** for TSA-treated and ATP-depleted cells, respectively, showing that the domain size increased to similar values upon perturbation of chromatin structure. **d** Chromatin mass density versus the number of loops per domain and the fiber persistence length calculated for the loop-cluster conformation under theta-solvent conditions. Highlighted areas represent the parameter subspace in agreement with previous studies. **e** Same as **d**, but for the globular and the blob conformation and thus without dependence on loop number

From the observed relaxation times, the radii of gyration of dynamic domains could be extracted according to Eq. 2 for loop-cluster topologies under theta-solvent conditions, for the same under good-solvent conditions, for globular conformations and for blobs. For untreated cells, this resulted for heterochromatin in 240 ± 6 nm and for euchromatin in 297 ± 9 nm (theta-solvent conditions, Fig. 5a; see Table 2 for a summary of the different conformations). Next, from fluorescence images we extracted chromatin densities in euchromatin of 91 ± 1 % and in heterochromatin of 156 ± 5 % of the mean nucleosome concentration of 100–140 µM [60, 72, 73] (Additional file 1: Supplementary Text, Fig. S7). In combination with a nucleosomal repeat length of 191 bp [72, 74], this enabled us to transform the domain volume determined from the radius of gyration into genomic content (Additional file 1: Eq. S10): We obtained 700–1120 and 830–1160 kb for hetero- and euchromatin, respectively, for blobs and loop clusters under theta-solvent conditions, 1230–1830 and 1470–2050 kb for loop clusters under good-solvent conditions, and 710–1050 and 840–1170 kb for globules.

For the good-solvent loop-cluster topology, the genomic content of domains was significantly larger than the previously observed 500–1000 kb for subchromosomal domains/TADs [11, 14], i.e., the assumption of good-solvent conditions would lead to a pronounced overestimation of domain size. Accordingly, the loop-cluster conformation under theta-solvent conditions was considered for further analysis. For this description, only minor excluded volume effects are present and thus a high structural flexibility on the level of the chain of nucleosomes. The blob and the globular polymer conformation would fit the TAD genome content but not the experimental interaction data from the 5C and T2C analysis as discussed above.

The polymer models predict a confined movement of chromatin segments relative to the center of mass of a domain, which is stationary on the time scale under consideration. Using the relaxation times obtained for the theta-solvent model, we calculated the MSD curves of a genomic site in euchromatin and heterochromatin (Fig. 5b), which clearly showed confinement of translocations and agreed well with experimental ones extracted directly from ACFs of exemplary measurements in euchromatin and heterochromatin according to Additional file 1: Eq. S83. Furthermore, the calculated MSDs corresponded well with previous studies of chromatin translocations [38–40, 72] and thus confirm our approach.

### Hyperacetylation and ATP depletion differentially affect chromatin dynamics and alter the radius of gyration of domains

Chromatin hyperacetylation due to TSA treatment of the cells slowed down chromatin relaxation, as apparent from a similarly increased radius of gyration at peripheral (*R*_*g*_ = 362 ± 14 nm) and central nuclear positions (*R*_*g*_ = 368 ± 15 nm) under theta-solvent conditions (Fig. 5c; Table 2). With a homogeneous nucleosome concentration of 100–140 µM, the genomic size of dynamic domains was 1650–2610 kb (Table 2), i.e., twofold larger than in untreated cells. This corroborates the view that hyperacetylation induces a larger-scale rearrangement of chromatin toward a more uniform conformation [70, 71] and the notion of discriminable compact and passive domains [56] whose differences vanish upon TSA treatment.

For ATP-depleted cells, radii of gyration increased to 367 ± 21 nm at peripheral and 356 ± 18 nm at central nuclear positions (Fig. 5c; Table 2). We obtained 2680–4100 and 1430–2160 kb for peripheral and central positions, respectively, when using the same mean nucleosome concentrations as for untreated cells. This suggests that in contrast to hyperacetylation, ATP depletion affects euchromatin and heterochromatin differentially as reflected by the increased heterogeneity in the images possibly due to agglutination of domains and increased packing density of nucleosomes.

### Local compaction of chromatin is determined by its flexibility, mass density and topology

To characterize the organization of the chromatin fiber into domains, a set of structural and physical parameters is required: the persistence length, the mass density and, in the case of looping, the number of loops per domain. We found that only certain combinations of the properties comply with the observed radius of gyration and genomic content. Figure 5d shows the relationship of number of loops per domain, chromatin persistence length and linear mass density computed for hetero- and euchromatin for loop clusters under theta-solvent conditions using Eq. 1 and a nucleosomal repeat length of 191 bp [72, 74]. The encircled area covers the parameter range compatible with previous knowledge [27, 74–77], i.e., a mass density of 0.5–6 nucleosomes/11 nm, a persistence length of 10–200 nm and up to 20 loops. A possible chromatin conformation with 9 loops per domain has a mass density of 4.5 nucleosomes/11 nm and a persistence length of 110 nm for euchromatin and 5.5 nucleosomes/11 nm and 100 nm for heterochromatin in very good agreement with Knoch et al. [53]. For a globular domain structure, the relation of persistence length and linear mass density computed for hetero- and euchromatin is depicted in Fig. 5e. Again, the marked area highlights the accessible part of parameter space and reveals a range of possible combinations, e.g., a mass density of 4.5 nucleosomes/11 nm and a persistence length of 55 nm for euchromatin and 5.5 nucleosomes/11 nm and 45 nm for heterochromatin. For both examples, the heterochromatin fiber would be more compacted but also locally more flexible. In contrast, for a blob-like domain structure, the relation of persistence length and mass density (Fig. 5e) does not overlap with previously obtained values, i.e., a purely generically formed chain-of-blob topology does not provide enough topological compaction. Thus, only the globule and the loop-cluster model agree with our observations for domain size and genomic content and only the latter with the 5C and T2C data.

Comparison of Fig. 5d with Fig. 1a, b showed that the large number of loops found for the ~1-Mb domains matched well with a persistence length of ~ 100 nm when assuming a mass density of ~4 nucleosomes/11 nm. Thus, FCS dynamics measurements allowed to detect dynamically independent subchromosomal domains, whereas 5C and T2C data allowed to detect topologically independent domains, and identifying them with each other enabled us to extract their size, genomic content, topology and average physical properties of the underlying chromatin fiber.

### Local chromatin dynamics determine genome accessibility

From the initial linear increase in the MSD (Fig. 5b), an apparent diffusion coefficient of ~0.1 µm^2^s^−1^ of chromatin segments could be extracted with a segment concentration of 10^4^–10^5^ µm^−3^ (Fig. 5d, e). From these parameters, a frequency of collisions with other sites could be estimated for a given genomic site inside a topological domain [78]: Intradomain collisions occur at a rates of ~100 collisions/s, whereas interdomain collisions are at least 100-fold less frequent. Therefore, contacts between genomic sites showing up in 3C-derived methods must be physically stable and long-lived enough to not be disrupted by the rapid local movements of the chromatin fiber, rendering stable looping a highly probable mechanism of domain formation.

**Fig. 6:**
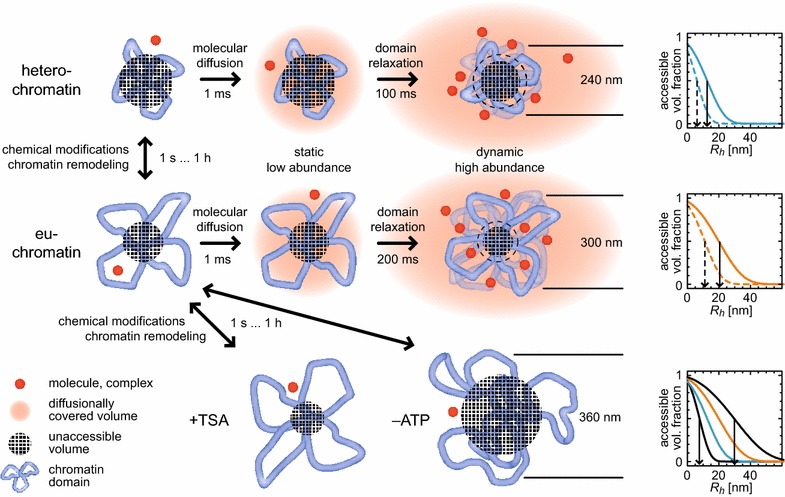
Static and dynamic chromatin domain accessibility. Model for domain accessibility of heterochromatin, euchromatin, TSA-treated and ATP-depleted chromatin. A certain volume fraction of the domain (*blue*) is not accessible (*checkered*) for soluble factors (*red*) due to steric hindrance. The domain is virtually static on time scales long enough for a single molecule to roam the domain volume by diffusion (~1 ms; *light red*), resulting in an unaccessible volume significantly larger than the net fiber volume. The single molecule is highly unlikely to return to a previously visited domain so that it only senses the static, snapshot-like unaccessible volume. Thus, accessibility for low-abundance molecules is defined by the apparently static conformation during the ms passage time. This effect is more pronounced for compact heterochromatin than for open euchromatin. On the time scale of domain reorganization (~100–200 ms), molecules can search different domain areas such that the effectively unaccessible volume decreases toward the net fiber volume (including ‘classical’ excluded volume effects). Accordingly, high-abundance molecules effectively sense a significantly higher accessible volume, i.e., accessibility depends on molecular concentration in addition to a binding reaction itself. Moreover, it is determined by the size of the molecule or complex (*arrows* in 1D plots), confirming previous findings on static chromatin accessibility. Thus, formation of domains consisting of dynamic loops provides an additional degree of freedom to differentially regulate chromatin accessibility. Chemical modifications and chromatin remodeling processes take place on significantly longer time scales so that access for required molecules can be regulated by domain and loop dynamics

The confined diffusion of chromatin segments (Fig. 5b) translates into pronounced volume fluctuations of the domains on the time scale of the observed relaxation times. The volume fluctuations are of the same order of magnitude as the volume itself, i.e., in the order of 0.1 µm^3^ (Additional file 1: Eq. S9). The time it takes soluble factors to cross a volume of the size of the domains by diffusion is around a few ms and much shorter than the relaxation time on the 100 ms time scale. Thus, the short-term accessibility of the domains for a single molecule is given by the statically occupied volume (Fig. 6). Many lacunae and corrals in the chromatin environment [42, 79] are devoid of scarce factors, so that locally, their effective concentration can be significantly smaller than the mean. For abundant molecules or complexes, however, it is defined by the fluctuation-induced maximum accessible volume. Thus, domains are adiabatically replenished to the mean concentration with molecules or complexes except for the net chromatin volume. Therefore, diffusion-limited reactions such as transcription factor binding to DNA are expected to display a more than linear dependence on factor concentration, in contrast to the case of soluble binding partners [78].

We calculated the accessible volume fraction according to Additional file 1: Eq. S74, S75 for euchromatin and heterochromatin as well as for TSA-treated cells, assuming both static and fluctuating domain sizes (Fig. 6). The accessibility limit, i.e., the molecular radius, for which accessibility was reduced to 50 %, was approximately twofold larger for dynamic domains than for static ones. Assuming an effective chromatin fiber diameter of 14 nm and a mass density of 1.6 nucleosomes/11 nm, the limit was 5 and 10 nm for heterochromatin, 10 and 20 nm for euchromatin, and 15 and 30 nm for TSA-treated cells for low- and high-abundance particles, respectively. This agreed well with previous results on chromatin accessibility [42, 71, 80] and showed that the fluctuations of the domains provide differential genome access in nonlinear dependence on particle size and concentration.

## Discussion

The results presented here provide a missing link between chromatin organization maps that reveal the subchromosomal domain structure at steady state from 3C-type analyses and the dynamic properties of these compartments measured here by FCS. The 3C-derived methods such as 5C, Hi-C or T2C as well as light microscopy measurements by fluorescence in situ hybridization/FISH [1, 7, 11, 14, 29, 81] yield more or less direct information about the relation between genomic and spatial distance in steady state. These have been used to evaluate physical models of three-dimensional chromatin organization [5, 29, 75–77, 82–85]. By applying a simple peak detection algorithm to exemplary experimental 5C and T2C data, the presence of loops and loop clusters is apparent, corroborating previous models and findings. From our analysis, we conclude that the highly dynamic nature of domains observed in our study provides an additional constraint on three-dimensional modeling of chromatin structure for 3C-type data: A high contact probability can only result from sufficiently stable physical contact between two loci, otherwise the pronounced fluctuations would effectively segregate them. We estimate that the lifetime of chromatin interactions must exceed a few seconds, i.e., significantly longer than the observed relaxation time, to be detected by chromosome conformation capture techniques. Moreover, the frequently occurring intradomain collisions of genomic sites are not rate limiting for contact formation between them. So far, one could only conclude that the interactions persisted for a significant fraction of the cross-linking incubation time of a few minutes [86, 87]. To our knowledge, this aspect has not been considered previously for the interpretation of 3C-like data.

Chromatin dynamics have been studied mostly by time-lapse microscopy and tracking or bleaching of spatially defined loci [35, 36, 40, 41]. While the time dependence of the MSD derived in these experiments provides evidence for the existence of distinct topological domains, it is difficult to draw quantitative conclusions on the underlying chromatin structure, especially on the time scale below one second. On the other hand, with our FCS-based methods we detected characteristic chromatin domain relaxation times in the order of 100 ms from measurements of the nuclear H1-EGFP signal (as well as of chromatin-incorporated core histones H2A and H2B). Furthermore, we developed an analytical Rouse–Zimm-based model that allows to derive polymer features from these data. Different conformations with topologies ranging from generically formed blobs via crumpled or fractal globules to loop-cluster/rosette formations can be represented to derive corresponding physical properties like persistence length and fiber density. In conjunction with the 5C/T2C analyses, we conclude that the dynamics of topological domains are best described by a clustered loop model in a theta solvent with radii of gyration of the domains of ~300 nm in euchromatin and ~240 nm in heterochromatin and a genomic content of ~0.8–1.2 Mb in the unperturbed state. We suggest to assign these domains to previously reported subchromosomal domains [15, 16] or TADs [11, 14], which have emerged as general pattern for chromatin organization in vertebrates [1, 3] and have been further confirmed by recent low-noise high-resolution T2C data [53]. They feature a typical size of ~1 Mb. Our data are in excellent agreement with previous studies that tracked chromatin foci [38–40, 72] and with persistence lengths and mass densities inferred from other studies [5, 27, 74–77]. We conclude that our observations are an independent and methodologically complementary quantitative evidence for dynamically and topologically independent domains that define both structural and dynamic properties of chromatin on the 1 Mb scale. In TSA-treated cells, euchromatin and heterochromatin become indistinguishable and both domain volume and genomic content increase, indicating a significant rearrangement of domains possibly owing to alternative remodeling following transcription and replication. In ATP-depleted cells, however, chromatin becomes more aggregated and both domain volume and genomic content increase, here possibly due to arrested transcription and chromatin remodeling.

Physical interactions between genomic loci via chromatin loops are important for the repression and activation of genes in the three-dimensional nuclear environment [4, 21, 23]. While the stability of loops is crucial for the robustness of gene expression patterns, plasticity and potential of domains for reorganization are key for gene up- or down-regulation in response to cellular stimuli [24, 35]. The highly dynamic nature of chromatin on the size scale of up to 1 Mb observed here with a typical locus spatially fluctuating by ~100 nm within ~100 ms facilitates fast rearrangement of three-dimensional topologies. In addition, as depicted in Fig. 6, it increases the effective chromatin accessibility, in good quantitative agreement with previous results: More compact heterochromatic domains have a larger unaccessible volume fraction than more open euchromatic ones. This effect additionally depends on the size of the molecules or complexes trying to access the genome [42, 71, 88]. Molecular diffusion is fast enough to roam a complete domain within few milliseconds, during which the domain itself appears static. Relaxation of domains in the 100 ms range affects genome access in a nonlinear protein concentration-dependent manner: Highly abundant molecules at several 100 nM concentrations ‘fill’ the fluctuating domain so that a larger volume fraction than for a static TAD becomes adiabatically accessible. In contrast, for low-abundance molecules encounters with specific loci within a domain are not only diffusion limited, but further impeded by transient occlusion of binding sites. They sense a higher inaccessible volume fraction. As a result, domain dynamics introduce an additional factor for nuclear target search. The concentration-dependent differential accessibility of this process leads to largely different search times as compared to a static chromatin network. Furthermore, it allows of locus-specific variations as relaxation times between heterochromatin and euchromatin are different and additionally dependent on reversible chromatin modifications like the TSA-induced hyperacetylation. Thus, by integrating the structural features of chromatin domains with their dynamic properties we reveal an additional regulatory layer for target search processes in the nucleus that may contribute to establishing cell-type-specific gene expression programs.

## Conclusions

In this study, we present a missing link between chromatin organization maps that reveal the subchromosomal domain structure at steady state from 3C-type analyses and the dynamic properties of these compartments measured here by FCS. Both 5C/T2C and FCS results suggest that chromatin is organized into topologically and dynamically independent domains of ~300 nm radius in euchromatin and ~240 nm in heterochromatin and a genomic content of ~0.8–1.2 Mb, confirming numerous previous results. Loops/loop clusters as domain-forming features are required to match the measured level of compaction and the observed features of 5C/T2C data. In addition to the structural aspects, the dynamics of domains in different epigenetic states propose that the regulation of chromatin accessibility for soluble factors displays a significantly stronger dependence on factor concentration than search processes within a static network.

## Methods

### Cell culture

The plasmid vector with the autofluorescent histone H1.0-GFP was constructed as described [89]. The human histone gene for H1.0 (Gene bank M87841) was amplified by PCR and inserted into the SalI–BamHI site of the promoterless plasmid pECFP-1 (Clontech, Mountain View, CA, USA). The HindIII fragment of simian virus 40 (SV40) was inserted in reverse direction into the HindIII site of the multiple cloning site of pECFP-1, and the ECFP sequence was replaced with EGFP. The resulting construct pSV-HIII-H1.0-EGFP expresses a 440-amino-acid fusion protein from the early SV40 promoter and consists of the human H1.0 gene, a 7-amino-acid linker and the C-terminal EGFP domain. This plasmid was introduced into MCF7 cells with Lipofectamin (Life Technologies, Carlsbad, CA, USA), and a stable monoclonal cell line was selected with 500 µg/ml G418 (Life Technologies). H1.0-expressing cells as well as non-transfected MCF7 cells were grown in RPMI 1640 (Life Technologies) supplemented with 10 % FCS in a humidified atmosphere under 5 % CO_2_ at 37 °C. HeLa cells expressing H2B–mCherry stably and H2A–EGFP transiently were made as described elsewhere [90].

For microscopy, cells were allowed to attach for at least 24 h in Nunc LabTek chambered coverglasses (Nalge Nunc, Rochester, NY, USA) or in MatTek glass-bottom dishes (MatTek, Ashland, MA, USA) before the experiments. For TSA treatment, cells were allowed to attach for at least 24 h in chambered coverglasses and then incubated with 100 ng/ml TSA (Sigma-Aldrich, St. Louis, MO, USA) for 15–20 h before the experiments. For Na-azide treatment, cells were allowed to attach for at least 24 h in chambered coverglasses and then incubated with 10 mM Na-azide for 20 min. Experiments were then performed within 40 min.

### Fluorescence microscopy

Confocal fluorescence microscopy images, FRAP image series, CP data, point FRAP data and FCS data were acquired with a Leica TCS SP2 AOBS FCS and with a Leica TCS SP5 AOBS FCS (Leica Microsystems, Mannheim, Germany) equipped with a 63×/1.2NA water immersion lens or with a Zeiss LSM 510 ConfoCor2 system (Carl Zeiss AIM, Jena, Germany) equipped with a 40×/1.2NA water immersion lens. For H1-EGFP, we used the 488 nm line of an Argon laser for excitation and a detection band-pass window of 500–550 nm. For imaging, photomultiplier tubes were used. For CP, point FRAP and FCS, avalanche photodiode single-photon counting detectors were used. Live cells were maintained at 37 °C on the microscopes using either a PeCon stage heating system (PeCon, Erbach, Germany), a Life Cell Imaging stage heating system (LCI, Seoul, South Korea) or an EMBL incubation box (EMBL-EM, Heidelberg, Germany).

### Imaging FRAP, point FRAP, CP

For imaging FRAP, a rectangular strip bleach region was defined. Acquisition of 10 prebleach images (time resolution 0.6 s) was followed by two bleach frames, 10 postbleach images (time resolution 0.6 s) and additional 40 postbleach images (time resolution 6 s). The data were then processed as described elsewhere [91, 92] to yield the mean intensity recovery curve integrated over the bleach region. This was then fitted with Additional file 1: Eq. S92, resulting in three different fractions, a diffusion coefficient and a dissociation rate. Alternatively, an average projection along the direction of the longer dimension of the bleach strip was plotted as profile along the other direction for all time points studied. Appropriate normalization steps [64, 92] (Additional file 1: Fig. S11) yielded profile plots that were then fitted with Additional file 1: Eq. S91 to yield an apparent diffusion coefficient.

Point FRAP and CP data were acquired as described elsewhere [43, 93, 94]. CP data were fitted with Additional file 1: Eq. S93 to yield two independent dissociation rates and corresponding fractions. Point FRAP data were fitted as described in Im et al. [43], however with two binding states.

### Fluorescence correlation spectroscopy

FCS data were acquired at cellular positions selected in confocal images for 30–60 s. A frequently encountered problem of FCS, especially in living samples, is slow but pronounced signal fluctuations, e.g., due to bulk photobleaching [43, 93–95] (Additional file 1: Fig. S8). Fluctuations contribute to the resulting correlation function (CF) weighted with the square of their brightness so that often slow fluctuations obscured completely the contributions from single diffusing molecules and rendered a further evaluation impossible. To overcome this obstacle, raw fluorescence intensity traces were saved to disk and then processed using the FluctuationAnalyzer software [90] written in our laboratory in C++ and LabVIEW (National Instruments, Austin, TX, USA) that used a local average approach where the CF is calculated over a small time window Θ and subsequently averaged over the complete length *T* according to

4 $$\begin{aligned} G_{kl} \left( {t^{\prime},\tau } \right) = \frac{{\left\langle {\delta F_{k} \left( t \right)\delta F_{l} \left( {t + \tau } \right)} \right\rangle_{{t^{\prime},\varTheta }} }}{{\left\langle {F_{k} \left( t \right)} \right\rangle_{{t^{\prime},\varTheta }} \left\langle {F_{l} \left( t \right)} \right\rangle_{{t^{\prime},\varTheta }} }}\quad {\text{with}}\quad \left\langle \ldots \right\rangle_{{t^{\prime},\varTheta }} = \frac{1}{\varTheta }\int\limits_{{t^{\prime}}}^{{t^{\prime} + \varTheta }} {{d}t \ldots } \;, \\ G_{kl} \left( \tau \right) = \left\langle {G\left( {t,\tau } \right)} \right\rangle \,\quad {\text{with}}\quad \left\langle \ldots \right\rangle = \left\langle \ldots \right\rangle_{{0,{T}}} \;. \\ \end{aligned}$$

Here, *k*, *l* = 1, 2 represent the two available detection channels. For *k* = *l* = 1, 2, the autocorrelation function (ACF) of channel 1, 2 is obtained, whereas *k* = 1, *l* = 2 yields the cross-correlation function (CCF). A good yet subjective criterion for a proper choice of the window size is a smooth transition of the CF to zero. In a more systematic way, we fitted the data with appropriate model functions, Eq. 3, 5. When finding a range of window sizes where, e.g., the relaxation time obtained from the fit was independent of the window size, we selected a window size within the range. Otherwise, the data were not taken into consideration.

To fit FCS data of the diffusive fraction of histone molecules and of free EGFP, we used the standard fit function modeling free anomalous diffusion and fluorescent protein-like blinking [96]

5 $$G_{kl} \left( \tau \right) = \frac{1}{N}\left[ {1 - \varTheta_{T} + \varTheta_{T} \exp \left( { - \frac{\tau }{{\tau_{T} }}} \right)} \right] \cdot \left[ {1 + \left( {\frac{\tau }{{\tau_{D} }}} \right)^{\alpha } } \right]^{ - 1} \left[ {1 + \frac{1}{{\kappa^{2} }}\left( {\frac{\tau }{{\tau_{D} }}} \right)^{\alpha } } \right]^{{ - {1 \mathord{\left/ {\vphantom {1 2}} \right. \kern-0pt} 2}}}$$

where *N* is the number of molecules in the focal volume, Θ_*T*_ the fraction of molecules in a non-fluorescent state with lifetime *τ*_*T*_, $\tau_{D} = {{w_{0}^{2} } \mathord{\left/ {\vphantom {{w_{0}^{2} } {4D}}} \right. \kern-0pt} {4D}}$ the diffusion correlation time, α the anomaly parameter and $\kappa = {{z_{0} } \mathord{\left/ {\vphantom {{z_{0} } {w_{0} }}} \right. \kern-0pt} {w_{0} }}$ the ratio of axial and lateral focal radius. Fitting FCS data with a chromatin relaxation model is described above.

### Numerical modeling of chromatin conformations

For the visualization and for the analysis of static physical properties of chromatin, we simulated chains as beads occupying sites on a three-dimensional cubic lattice with a grid constant of *a* = 30 nm. Neighboring sites were connected by chain segments, and neighbors could occupy any of the surrounding 26 sites, resulting in a mean distance or bond length of $b = \sqrt 2 a = 42{\text{ nm}}$ corresponding to 2500 bp when assuming 60 bp/nm or 3.5 nucleosomes/11 nm and 195 bp nucleosomal repeat length. The grid constant is set to an assumed fiber diameter of 30 nm. Double occupancy of sites is suppressed to ensure self-avoidance of the chain. In general, chains were modeled as a sequence of loops and linear stretches. Properties such as radii of gyration were calculated according to the respective definition. Calculations were implemented in Python 3.3, and renderings were generated using the VPython module.

### Calculation of genomic contact probability maps

We calculated genomic contact probability maps for simulated chromatin conformation using Additional file 1: Eq. S25 and the algorithm described in the Additional file 1: Supplementary Text. Data were saved as matrices with a resolution of 2.5 kb. For the configurations used in Fig. 1d, e we used the following parameters:

- Figure 1d: theta-solvent loop-rosette conformation; lin(*x*)—linear stretch of *x* kb; dom(*y*)—domain of *y* kb consisting of a set of loops; loop(*z*)—looped stretch of *z* kb; loops with multiple numbers were varied synchronously in length and then averaged to generate variation in loop length. lin(100) – dom (1000) [loop(166) – loop(167) – loop(166) – loop(167) – loop(166)] – lin(150) – dom (1300) [loop(100/125/150/175/200) – loop(95) – loop(90) – loop(85) – loop(120/145/170/195/220) – loop(150) – loop(125) – loop(115) – loop(160) – loop(150)] – lin(150) – dom(1000) [loop(185) – loop(120) – loop(95) – loop(120) – loop(235) – loop(245)] – lin(50) – dom(1100) [loop(138) – loop(160) – loop(95) – loop(170) – loop(160) – loop(183) – loop(128) – loop(68)] – lin(150)
- Figure 1e: globular conformation; lin(*x*)—linear stretch of *x* kb; dom(*y*)—domain of *y* kb consisting of a globular stretch; glob(*z*)—globular stretch of *z* kb. lin(100) – dom(1000) [glob(1000)] – lin(150) – dom(1300) [glob(1300)] – lin(150) – dom(1000) [glob(1000)] – lin(50) – dom(1100) [glob(1100)] – lin(150)

### Analysis of genomic contact probability maps

To detect peaks in the two-dimensional contact probability maps, both experimental and simulated data were imported into a software module written in LabVIEW. It allowed to interpolate data to a resolution of 2.5 kb and to symmetrize them. After manually selecting a domain region easily recognizable as square area of increased contact probabilities (Fig. 1a, b, d, e), the diagonal and its vicinity of ±30–75 kb (±12–30 data points of 2.5 kb) were removed. A one-dimensional average of a maximum and a mean projection (Additional file 1: Fig. S1) yielded a one-dimensional profile, to which a peak detection algorithm was applied based on parabolic fitting to continuous stretches of 30 kb (12 data points). Maxima above 80 % of the profile average were accepted as peak locations.

Then, local average projections in a 25- to 30-kb vicinity of each peak were calculated (Additional file 1: Fig. S1), to which the same peak detection algorithm was applied. Thus, for each peak detected in this way, a pair of genomic sites of high interaction probability could be obtained, corresponding to a loop base. Pairs detected in both directions featured higher recognition probability and were marked with black circles (Fig. 1a, b, d, e), and those detected with lower probability, i.e., only in one direction, were marked with gray circles. This approach corresponds to an effective thresholding of distances instead of using their values [97] justified by the dynamic nature of domains and is applied to non-corrected and smoothed data similar to Giorgetti et al. [28]. The binarization is especially robust against bias effects, which are not completely known even though corrections can be applied [98, 99].

## Additional file

**Additional file 1.** Additional documentation.
