# Supplementary material for: Dynamic properties of independent chromatin domains measured by correlation spectroscopy in living cells
Source: Epigenetics Chromatin. 2016 Dec 24;9:57. doi: 10.1186/s13072-016-0093-1 (PMC5192577; doi:10.1186/s13072-016-0093-1)
Supplement: Supplementary file 1 — Additional file 1. Additional documentation. [file 13072_2016_93_MOESM1_ESM.pdf]

Additional File 1: Additional Documentation for

Dynamic properties of independent chromatin domains measured by correlation spectroscopy in living cells

Malte Wachsmuth[1*], Tobias A. Knoch[2] and Karsten Rippe[3]

[1] Cell Biology and Biophysics Unit, European Molecular Biology Laboratory (EMBL), Meyerhofstrasse 1, D-69117 Heidelberg, Germany

[2] Biophysical Genomics Group, Dept. Cell Biology & Genetics, Erasmus Medical Center, Dr. Molewaterplein 50, NL-3015 GE Rotterdam, The Netherlands

[3] Research Group Genome Organization & Function, Deutsches Krebsforschungszentrum (DKFZ) & BioQuant, Im Neuenheimer Feld 280, D-69120 Heidelberg, Germany

[*] Correspondence: m.wachsmuth@gmx.net

Content

Supplementary Text

1. Static properties of polymers
2. Dynamic properties of polymers
3. Fluorescence correlation spectroscopy of polymer relaxation
4. Photobleaching experiments of chromatin-associated proteins
5. Determination of eu- and heterochromatin nucleosome concentration from images
6. Determination of nuclear solvent viscosity

Supplementary Figures

Supplementary Figure S1: Profile-based peak detection for genomic contact probability maps
Supplementary Figure S2: Structural analysis of 5C data
Supplementary Figure S3: Structural analysis of T2C data
Supplementary Figure S4: Multiscale properties of simulated domain topologies
Supplementary Figure S5: Experimental and simulated 5C data and domain structure
Supplementary Figure S6: Experimental and simulated T2C data and domain structure
Supplementary Figure S7: Image intensity analysis and classification
Supplementary Figure S8: FCS in the presence of photobleaching
Supplementary Figure S9: Confocal and light-sheet FCS on different scales
Supplementary Figure S10: FCS in fixed HeLa cells expressing H2A-EGFP
Supplementary Figure S11: Strip profile analysis of FRAP experiments
Supplementary Figure S12: Simulated FRAP experiments

Supplementary References

Supplementary Text

Static properties of polymers

1.1. The linear freely jointed chain

The freely jointed chain forms the basis to derive static and dynamic properties of polymers [1, 2]. Being a mere theoretical construct, it is well suited to describe more realistic models such as the Gaussian, the persistent/worm-like chain or the Kratky-Porod model.

The freely jointed chain is a linear chain of rigid rods of length b that are connected with completely flexible joints. Each segment i corresponds to the vector $\boldsymbol{u}_i$ of length l and the end-to-end vector is thus

$$\boldsymbol{R} = \sum_{i=1}^{N} \boldsymbol{u}_i, \quad |\boldsymbol{u}_i| = b. \tag{1}$$

The mean squared end-to-end distance is given as

$$\left\langle \boldsymbol{R}^2 \right\rangle = \left\langle \left(\sum_{i=1}^{N} \boldsymbol{u}_i \right)^2 \right\rangle = \sum_{i=1}^{N} \left\langle \boldsymbol{u}_i^2 \right\rangle + \sum_{i \neq j} \underbrace{\left\langle \boldsymbol{u}_i \boldsymbol{u}_j \right\rangle}_{=0} = Nb^2 = Lb \tag{2}$$

where b is the so-called Kuhn segment length and L the total contour length. Any hydrodynamic, electrostatic or excluded volume effects and interactions are disregarded.

1.2. The persistent or worm-like chain, Kratky-Porod model

These simplifications hold as well for continuous flexible or persistent chains where b becomes related to the elastic properties of the chain. It can be shown that for a continuous Markov chain, the average angle between segments at a distance s obeys

$$\left\langle \cos\Theta(s+s') \right\rangle = \left\langle \cos\Theta(s) \right\rangle \left\langle \cos\Theta(s') \right\rangle \quad \Rightarrow \quad \left\langle \cos\Theta(s) \right\rangle = \exp\left(-\frac{s}{l_p} \right), \tag{3}$$

i.e., the chain has an exponentially decaying directional memory, whose characteristic length l_p is the so-called persistence length. The Kratky-Porod model relates this so far empirical number to the bending elasticity or the energy required to bend it into a certain curvature.

In the continuous model, tangential vectors instead of segments are used to calculate the the mean squared end-to-end distance:

$$\begin{aligned} \left\langle \boldsymbol{R}^2 \right\rangle &= \int_0^L ds \int_0^L ds' \left\langle \boldsymbol{u}(s) \boldsymbol{u}(s') \right\rangle = 2 l_p \left[\frac{L}{l_p} - 1 + \exp\left(-\frac{L}{l_p} \right) \right] \\ &= \begin{cases} L^2 & L < l_p, \\ 2 L l_p & L > l_p. \end{cases} \end{aligned} \tag{4}$$

The second case shows that in the long contour length limit, the worm-like chain behaves like a freely jointed chain and the Kuhn segment length relates to the persistence length as

$$b = 2l_p. \tag{5}$$

1.3. The Gaussian chain

If the direction of real or virtual (Kuhn) segments of a polymer are stochastically independent or feature an exponentially decaying correlation the central limit theorem allows to describe the end-to-end distribution of the chain with a Gaussian function

$$\psi(\boldsymbol{R}) = \left(\frac{3}{2\pi\langle \boldsymbol{R}^2\rangle}\right)^{3/2} \exp\left(-\frac{3\boldsymbol{R}^2}{2\langle \boldsymbol{R}^2\rangle}\right). \tag{6}$$

In case segments are completely independent at distances larger than b, the polymer is a Markov chain, and for points chosen equally distributed along the chain and sufficiently far apart the pair-wise distance distributions are independent and Gaussian, too, i.e., subchains behave in the same way.

Thus, both freely jointed and worm-like chains can be described as Gaussian chains of virtual beads on a string that have a Gaussian distance distribution. Their spacing usually does not match the chemical bond length but is chosen such that their static and dynamic properties match the real polymer. This serves as a basis to derive the dynamic properties of polymers in the Rouse and Zimm models.

1.4. Properties of Gaussian chains

The most obvious characteristic size of a coiled linear chain is the mean squared end-to-end distance, which can also be identified with the correlation length of the coil and which defines the order of magnitude of all characteristic size properties.

The radius of gyration is defined as the mean squared mutual distance of all segments or equivalently the mean squared distance of the segments from the center of mass:

$$\begin{aligned} R_g^2 &= \frac{1}{2N^2}\left\langle \sum_{i=1}^{N}\sum_{j=1}^{N} \boldsymbol{R}_{ij}^2 \right\rangle = \frac{1}{N}\left\langle \sum_{k=1}^{N}(\boldsymbol{R}_k - \boldsymbol{R}_{cm})^2 \right\rangle \\ &= \frac{1}{6}\langle \boldsymbol{R}^2\rangle = \frac{Nb^2}{6} \quad \text{with} \quad \langle \boldsymbol{R}_{ij}^2\rangle = |i-j|b^2. \end{aligned} \tag{7}$$

It is experimentally accessible with scattering methods [3] and determines internal relaxation processes.

The hydrodynamic radius corresponds to the radius of a sphere with the same diffusion coefficient as the coiled chain. It is defined as

$$\begin{aligned} R_h^{-1} &= \frac{1}{N^2}\left\langle \sum_{i=1}^{N}\sum_{j=1}^{N} \frac{1}{|\boldsymbol{R}_{ij}|} \right\rangle, \\ R_h^2 &= \frac{3\pi}{128}\langle \boldsymbol{R}^2\rangle = \frac{3\pi}{128}Nb^2 \quad \text{with} \quad \left\langle \frac{1}{|\boldsymbol{R}_{ij}|}\right\rangle = \sqrt{\frac{6}{\pi}}\frac{1}{|i-j|b}. \end{aligned} \tag{8}$$

The variance of the mean squared end-to-end distance is of the same order as the end-to-end distance itself

$$\frac{\left\langle \boldsymbol{R}^4 \right\rangle - \left\langle \boldsymbol{R}^2 \right\rangle^2}{\left\langle \boldsymbol{R}^2 \right\rangle^2} = \frac{2}{3} . \tag{9}$$

The same applies to the radius of gyration and provides an estimation for size fluctuations in different geometries and topologies.

According to the central limit theorem, the density distribution of segments around the center of mass obeys a Gaussian distribution. In a continuous approximation of the chain, the density distribution and the occupied volume are

$$\begin{aligned} \psi(\boldsymbol{r}) &= \exp\left[-\frac{3(\boldsymbol{r} - \boldsymbol{R}_{cm})^2}{2R_g^2} \right], \\ V &= \int d^3 r\, \psi(\boldsymbol{r}) = \left(\frac{2\pi}{3} R_g^2 \right)^{3/2} \approx 3.03 R_g^3 , \end{aligned} \tag{10}$$

the latter allowing to determine average densities or concentrations. Applying Equation (9) to the definition of the radius of gyration results in an estimation of the upper volume limit induced by fluctuations:

$$\frac{V + \delta V}{V} = 1 + \frac{3}{2} \cdot \sqrt{\frac{2}{3}} = 1 + \sqrt{\frac{3}{2}} \approx 2.22 . \tag{11}$$

1.5. Real polymers: interactions between segments

A real polymer is always in a balance between an excluded volume effect represented by a hard and sharp repulsive potential and a weak attractive interaction (van der Waals, electrostatic or entropic) that depends strongly on the solvent conditions [1]: in a 'good solvent', the excluded volume interaction dominates and the polymer coil is swollen compared to the ideal chain. In a 'theta solvent', repulsive and attractive interactions compensate each other such that the chain behaves like an ideal self-permeating polymer. The last case is a 'poor solvent' where the attractive potential dominates, the polymer collapses and it assumes a globular state. This concept was first introduced by Kuhn and Flory [4, 5] and subsequently refined using perturbation calculations and the concept of renormalization groups. In order to distinguish the different cases one can define a swelling coefficient

$$\alpha^2 = \frac{R_g^2}{R_{g,0}^2} \begin{cases} > 1 & \text{good solvent/swollen} \\ = 1 & \Theta \text{ solvent/ideal} \\ < 1 & \text{poor solvent/globular} \end{cases} \tag{12}$$

as the ratio of the radii of gyration of the real and the ideal polymer. It depends on the chain length, resulting in a different dependence of the mean squared distance of two segments [2]:

$$\left\langle \boldsymbol{R}_{nm}^2 \right\rangle = \left(|n-m|^\nu b\right)^2 \quad \text{with} \quad \begin{cases} \nu = 3/5 & \text{good solvent,} \\ \nu = 1/2 & \text{theta solvent,} \\ \nu = 1/3 & \text{poor solvent.} \end{cases} \tag{13}$$

For a discrete to a continuous parametrization of the chain, the radius of gyration is

$$R_g^2 = \frac{b^2}{2N^2}\int_0^N dn \int_0^N dm |m-n|^{2\nu} = \frac{N^{2\nu} b^2}{(2\nu+1)(2\nu+2)} = \begin{cases} \dfrac{N^{6/5}b^2}{176/25} \approx \dfrac{N^{6/5}b^2}{7.04} & \nu = \dfrac{3}{5}, \\ \dfrac{Nb^2}{6} & \nu = \dfrac{1}{2}, \\ \dfrac{N^{2/3}b^2}{40/9} \approx \dfrac{N^{2/3}b^2}{4.44} & \nu = \dfrac{1}{3}. \end{cases} \tag{14}$$

For the distance distribution of two segments or beads

$$\psi_{nm}(r) = \psi_{nm}\left(|\boldsymbol{R}_n - \boldsymbol{R}_m|\right) \tag{15}$$

again, the three cases must be treated separately. For the ideal and the globular chain, the compensation and overcompensation, respectively, of the repulsive by the attractive interaction allows to consider the polymer as soft chain [6] and to describe the segment distances as Gaussian distributions when neglecting correction terms of order r^2 and higher [7]:

$$\psi_{nm}(r) = \begin{cases} \left(\dfrac{3}{2\pi|n-m|b^2}\right)^{3/2} \exp\left(-\dfrac{3r^2}{2|n-m|b^2}\right) & \nu = \dfrac{1}{2}, \\ \left(\dfrac{3}{2\pi|n-m|^{2/3}b^2}\right)^{3/2} \exp\left(-\dfrac{3r^2}{2|n-m|^{2/3}b^2}\right) & \nu = \dfrac{1}{3}, \end{cases} \tag{16}$$

which yield the mean squared segment distances according to $\left\langle \boldsymbol{R}_{nm}^2 \right\rangle = \int d^3r\, r^2 \psi_{nm}(r)$.

For the swollen chain, however, we apply the following conceptual constraints and approximations [1, 2]:

(i) it must be normalized,

(ii) it must yield the above-mentioned mean squared distance, Equation (14),

(iii) $\psi_{nm}(r) = \begin{cases} r^{5/18} & r < |n-m|^\nu b, \\ \exp\left(-cr^{5/2}\right) & r > |n-m|^\nu b, \end{cases}$

(iv) $\psi_{nm}(r) = f\left(\dfrac{r}{|n-m|^{3/5} b}\right)$,

resulting in the numerical approximation

$$\psi_{nm}(r) \approx \frac{0.29}{\left(|n-m|^{3/5} b\right)^{3.28}} r^{0.28} \exp\left[-1.22\left(\frac{r}{|n-m|^{3/5} b}\right)^{5/2}\right]. \tag{17}$$

1.6. Blobs as globular substructures of the chain

The emergence of globular substructures of a polymer chain in theta- or good-solvent conditions such as the chromatin fiber can be explained as the formation of a chain of so-called 'blobs' when the system is at semi-dilute concentrations [8, 9], i.e., when the concentration is too high for individual molecules to be considered isolated, see also Section 2.9 for further details. The blobs themselves have the same static and dynamic properties of the whole molecule under highly diluted conditions. They can be described as shorter independent linear chains in theta- or good-solvent conditions.

1.7. Properties of circular polymers

In addition, globular substructures may result from the formation of of loops. Therefore, here we describe the static properties of cicrular polymers following the same scheme as above [1, 10, 11]. However, only the ideal and the swollen chain are considered because under poor-solvent conditions, it inherently forms globular structures. The spatial distance distribution of two segments of a ring is composed of both possible paths, resulting in an effective segment distance along the chain

$$\mu(m,n,N) = \frac{|m-n|(N-m+1)}{N}, \quad 0 \le m,n \le N \tag{18}$$

and a mean squared distance of

$$\left\langle \boldsymbol{R}_{nm}^2 \right\rangle = \begin{cases} \mu b^2 & \nu = 1/2, \\ \mu^{6/5} b^2 & \nu = 3/5. \end{cases} \tag{19}$$

This yields a radius of gyration of

$$R_g^2 = \frac{b^2}{2N^2} \int_0^N dn \int_0^N dm\, \mu(m,n,N)^{2\nu} = \begin{cases} \dfrac{Nb^2}{12} = \dfrac{R_{g,\mathrm{lin}}^2}{2} & \nu = \dfrac{1}{2}, \\ \dfrac{N^{6/5} b^2}{16.70} \approx \dfrac{R_{g,\mathrm{lin}}^2}{2.37} & \nu = \dfrac{3}{5}. \end{cases} \tag{20}$$

1.8. Branched polymers: stars and rosettes

For loops and even more for branched polymers, a mean squared end-to-end distance cannot be determined and the mean squared pair-wise segment distance, i.e., the radius of gyration, is used to compare macromolecules of different topologies. Since in branched polymers, the density grows with increasing branching ratio, an excluded volume effect should also be more prominent.

The radius of gyration is calculated as the sum of the mean squared segment distance within branches and between branches. We restrict the previously established approach [12-14] to a single multiple branching point and extend it from ideal to swollen molecules. This yields for the

(i) ideal star-like branched linear chain (theta-solvent conditions with $\nu = 1/2$) the contributions

$$f \frac{b^2}{2N^2} \int_0^{N/f} dn \int_0^{N/f} dm |m-n| = \frac{Nb^2}{6f^2} \quad \text{within branches,}$$

$$f(f-1)\frac{b^2}{2N^2} \int_0^{N/f} dn \int_0^{N/f} dm (m+n) = \frac{Nb^2(f-1)}{2f^2} \quad \text{between branches} \tag{21}$$

$$\Rightarrow \quad R_g^2 = \frac{Nb^2}{6}\left(\frac{3f-2}{f^2}\right),$$

(ii) ideal rosette-like branched circular chain (theta-solvent conditions with $\nu = 1/2$) the contributions

$$f \frac{b^2}{2N^2} \int_0^{N/f} dn \int_0^{N/f} dm\, \mu(m,n,N/f) = \frac{Nb^2}{12f^2} \quad \text{within branches,}$$

$$f(f-1)\frac{b^2}{2N^2} \int_0^{N/f} dn \int_0^{N/f} dm [\mu(m,0,N/f) + \mu(n,0,N/f)] = \frac{Nb^2(f-1)}{6f^2} \quad \text{between branches} \tag{22}$$

$$\Rightarrow \quad R_g^2 = \frac{Nb^2}{12}\left(\frac{2f-1}{f^2}\right),$$

(iii) swollen star-like branched linear chain (good-solvent conditions with $\nu = 3/5$) using the same approach as above

$$R_g^2 = \frac{N^{6/5}b^2}{7.04}\left(\frac{3.59f - 2.59}{f^{2.2}}\right), \tag{23}$$

(iv) swollen rosette-like branched circular chain (good-solvent conditions with $\nu = 3/5$)

$$R_g^2 = \frac{N^{6/5}b^2}{16.70}\left(\frac{1.92f - 0.92}{f^{2.2}}\right). \tag{24}$$

1.9. Contact probabilities of polymer segments

The chromatin fiber of a chromosome might be organized in an intricate manner including loops and clusters thereof, but the underlying single linear molecule allows to arrange the polymer segments sequentially. Depending on the topology, the effective contour length or distance σ between two segments with the indices m and n can be calculated using $|n-m|$ for linear and Equation (18) for circular stretches. As an example, segment m is connected to segment $n = m + k + p$ through a linear stretch of k segments and a stretch of p segments into a loop of L segments. The effective distance is thus $\sigma = k + \mu(p,0,L)$. Depending on the topology and the physical properties of the fiber as well as on the solvent conditions, the contact probability, defined as the probability that the two segments come as close as the contact distance d_c or closer to each other, is calculated using Equation (16) and (17):

$$p(\sigma,b,d_c)=4\pi\int_0^{d_c} dr\, r^2 \psi(r,\sigma,b),$$

$$\psi(r,\sigma,b)=\begin{Bmatrix} \left(\frac{3}{2\pi\sigma b^2}\right)^{3/2}\exp\left(-\frac{3r^2}{2\sigma b^2}\right) & \nu=\frac{1}{2} \\ \left(\frac{3}{2\pi\sigma^{2/3} b^2}\right)^{3/2}\exp\left(-\frac{3r^2}{2\sigma^{2/3} b^2}\right) & \nu=\frac{1}{3} \\ \frac{0.29}{\left(\sigma^{3/5}b\right)^{3.28}} r^{0.28}\exp\left[-1.22\left(\frac{r}{\sigma^{3/5}b}\right)^{5/2}\right] & \nu=\frac{3}{5} \end{Bmatrix} \Rightarrow$$

$$p(d_c,\sigma,b)=\begin{cases} \mathrm{erf}\left[\left(\frac{3d_c^2}{2\sigma b^2}\right)^{1/2}\right]-\frac{2}{\pi^{1/2}}\left(\frac{3d_c^2}{2\sigma b^2}\right)^{1/2}\exp\left(-\frac{3d_c^2}{2\sigma b^2}\right) & \nu=\frac{1}{2}, \\ \mathrm{erf}\left[\left(\frac{3d_c^2}{2\sigma^{2/3} b^2}\right)^{1/2}\right]-\frac{2}{\pi^{1/2}}\left(\frac{3d_c^2}{2\sigma^{2/3} b^2}\right)^{1/2}\exp\left(-\frac{3d_c^2}{2\sigma^{2/3} b^2}\right) & \nu=\frac{1}{3}, \\ 1-\Gamma_{\mathrm{inc,upper}}\left[1.312, 1.22\left(\frac{d_c}{\sigma^{3/5}b}\right)^{5/2}\right] & \nu=\frac{3}{5}. \end{cases} \quad (25)$$

With this, modelling of the topological sequence of linear, looped or clustered stretches enables to calculate intrachromosomal interaction maps as known e.g. from 3C, 4C, 5C, Hi-C or T2C studies [15-17].

Dynamic properties of polymers

1.10. Rouse model for a linear chain

In order to determine the dynamic properties of polymers, Rouse [1, 18] proposed to model the molecules as Gaussian chains of beads connected with springs. Their spacing b is the the Kuhn segment length, which does not necessarily correspond to the bond length between monomeric units but is determined by the physical properties of the molecule, i.e., the thermally induced root mean squared distance of neighboring beads sensing a harmonic potential U.

Then, for every segment n, the Langevin equation is given as

$$\frac{\partial \boldsymbol{R}_n(t)}{\partial t}=\sum_m \hat{\boldsymbol{H}}_{nm}\left(-\frac{\partial U}{\partial \boldsymbol{R}_m}+\boldsymbol{F}_m(t)\right)+\frac{k_B T}{2}\sum_m \frac{\partial}{\partial \boldsymbol{R}_m}\hat{\boldsymbol{H}}_{nm}. \quad (26)$$

In the Rouse model, the excluded volume interaction and the hydrodynamic interaction (represented by the mobility or Oseen tensor $\hat{\boldsymbol{H}}$) are neglected so that this case is often referred to as free-draining polymer. The Oseen tensor and the interaction potential are written as

$$\hat{\boldsymbol{H}}_{nm}=\frac{\boldsymbol{1}}{\gamma}\delta_{nm},\quad U=\frac{k}{2}\sum_{n=2}^{N}\left(\boldsymbol{R}_n-\boldsymbol{R}_{n-1}\right)^2 \quad \text{with} \quad k=\frac{3k_B T}{b^2}, \quad (27)$$

where γ stands for the friction coefficient, k for the entropic spring constant and $\boldsymbol{F}$ for the thermally induced random forces. With a transition from a discrete to a continuous model, the Langevin equation simplifies to

$$\gamma \frac{\partial \boldsymbol{R}_n}{\partial t} = k \frac{\partial^2 \boldsymbol{R}_n}{\partial n^2} + \boldsymbol{F}_n \tag{28}$$

with the boundary conditions

$$\left.\frac{\partial \boldsymbol{R}_n}{\partial n}\right|_{n=0} = \left.\frac{\partial \boldsymbol{R}_n}{\partial n}\right|_{n=N} = 0 \tag{29}$$

and the moments of the random forces

$$\langle \boldsymbol{F}_n(t) \rangle = 0, \quad \langle \boldsymbol{F}_n(t) \boldsymbol{F}_m(t') \rangle = 6\gamma k_B T \delta(n-m) \delta(t-t'). \tag{30}$$

An appropriate transform gives normal coordinates $\boldsymbol{X}_p$ according to

$$\boldsymbol{X}_p(t) = \frac{1}{N} \int_0^N dn \cos\left(\frac{p\pi n}{N}\right) \boldsymbol{R}_n(t), \tag{31}$$

for which the equations of motion are then written as

$$\gamma_p \frac{\partial \boldsymbol{X}_p}{\partial t} = -k_p \boldsymbol{X}_p + \boldsymbol{F}_p \tag{32}$$

where

$$\begin{aligned} &\gamma_0 = N\gamma, \quad \gamma_p = 2N\gamma \quad \text{for} \quad p = 1, 2, \ldots, \\ &k_p = \frac{6\pi^2 k_B T}{N b^2} p^2 \quad \text{for} \quad p = 0, 1, 2, \ldots \quad . \end{aligned} \tag{33}$$

For $p > 0$, a solution can be found employing the autocorrelation function of the normal coordinates:

$$\begin{aligned} &\langle \boldsymbol{X}_p(t) \boldsymbol{X}_q(0) \rangle = \delta_{pq} \langle \boldsymbol{X}_p^2(0) \rangle \exp\left(-\frac{t}{\tau_p}\right), \\ &\langle \boldsymbol{X}_p^2(0) \rangle = \frac{3k_B T}{k_p} = \frac{N b^2}{2\pi^2 p^2}, \\ &\tau_1 = \frac{\gamma_1}{k_1} = \frac{\gamma N^2 b^2}{3\pi^2 k_B T}, \quad \tau_p = \frac{\tau_1}{p^2}, \end{aligned} \tag{34}$$

i.e., they represent independent modes with exponentially decaying correlation functions.

For $p = 0$, on the other hand, one can show using the inverse transform

$$\boldsymbol{R}_n(t) = \boldsymbol{X}_0(t) + 2 \sum_{p=1}^{\infty} \boldsymbol{X}_p(t) \cos\left(\frac{p\pi n}{N}\right) \tag{35}$$

that the mean squared displacement (MSD) of the center of mass of the molecule is described by

$$\left\langle \left[\boldsymbol{X}_0(t) - \boldsymbol{X}_0(0) \right]^2 \right\rangle = 6Dt \quad \text{with} \quad D = \frac{k_B T}{N\gamma}. \tag{36}$$

It is worth noting that the end-to-end distance relaxation is dominated by the first mode. This mode corresponds to the rotation of the molecule and thus relates its relaxation time directly to the radius of gyration. However, especially the mass dependence of the diffusion coefficient D and of the relaxation times is contradictory to experimental observations mainly because the hydrodynamic interaction was disregarded so that the Rouse model serves as a good conceptual basis but must be adpated to experimental reality.

A readout that is well accessible experimentally for chromatin in the nucleus of a living cell in interphase is the relative movement of a segment with respect to the center of mass averaged over all segments, i.e., averaged over $n \in [0, N]$. The corresponding correlation function reads

$$\left\langle \boldsymbol{R}'_{\text{seg}}(t) \boldsymbol{R}'_{\text{seg}}(0) \right\rangle = 2 \sum_{p=1}^{\infty} \left\langle \boldsymbol{X}_p^2(0) \right\rangle \exp\left(-\frac{t}{\tau_p} \right), \tag{37}$$

i.e., all modes contribute to the averaged movement of the segments according to their amplitude. Likewise, the mean squared displacement of a segment again with respect to the center of mass and averaged over all segments can be obtained, which shows confined diffusion behaviour:

$$\left\langle \left[\boldsymbol{R}'_{\text{seg}}(t) - \boldsymbol{R}'_{\text{seg}}(0) \right]^2 \right\rangle = 4 \sum_{p=1}^{\infty} \left\langle \boldsymbol{X}_p^2(0) \right\rangle \left[1 - \exp\left(-\frac{t}{\tau_p} \right) \right] = 4 \sum_{p=1}^{\infty} \left\langle \boldsymbol{X}_p^2(0) \right\rangle - 2 \left\langle \boldsymbol{R}'_{\text{seg}}(t) \boldsymbol{R}'_{\text{seg}}(0) \right\rangle. \tag{38}$$

1.11. Rouse model for a ring

For a closed circular chain, the same approach is applied [19], however with different boundary conditions:

$$\boldsymbol{R}_0 = \boldsymbol{R}_n, \quad \left. \frac{\partial \boldsymbol{R}_n}{\partial n} \right|_{n=0} = \left. \frac{\partial \boldsymbol{R}_n}{\partial n} \right|_{n=N}. \tag{39}$$

The transform is rewritten as

$$\boldsymbol{X}_p(t) = \frac{1}{N} \int_0^N dn \cos\left(\frac{2p\pi n}{N} \right) \boldsymbol{R}_n(t) \tag{40}$$

and the parameters are

$$\begin{aligned} &\gamma_0 = N\gamma, \quad \gamma_p = 2N\gamma \quad \text{for} \quad p = 1, 2, \ldots, \\ &k_p = \frac{24\pi^2 k_B T}{Nb^2} p^2 \quad \text{for} \quad p = 0, 1, 2, \ldots, \end{aligned} \tag{41}$$

and the relaxation of the modes changes to

$$\left\langle \boldsymbol{X}_p^2(0) \right\rangle = \frac{3k_BT}{k_p} = \frac{Nb^2}{8\pi^2 p^2},$$
$$\tau_1 = \frac{\gamma_1}{k_1} = \frac{\gamma N^2 b^2}{12\pi^2 k_B T}, \quad \tau_p = \frac{\tau_1}{p^2}. \tag{42}$$

Thus, the relaxation modes of a circular polymer are the even modes of a linear chain of the same length. The same result as for a linear chain is obtained for the zero order mode, the translational diffusion. Also the averaged segment movement relative to the center of mass is given as position correlation function and mean squared displacement, respectively:

$$\left\langle \boldsymbol{R}'_{\mathrm{seg}}(t)\boldsymbol{R}'_{\mathrm{seg}}(0) \right\rangle = 2\sum_{p=1}^{\infty} \left\langle \boldsymbol{X}_p^2(0) \right\rangle \exp\left(-\frac{t}{\tau_p}\right),$$
$$\left\langle \left[\boldsymbol{R}'_{\mathrm{seg}}(t) - \boldsymbol{R}'_{\mathrm{seg}}(0)\right]^2 \right\rangle = 4\sum_{p=1}^{\infty} \left\langle \boldsymbol{X}_p^2(0) \right\rangle - 2\left\langle \boldsymbol{R}'_{\mathrm{seg}}(t)\boldsymbol{R}'_{\mathrm{seg}}(0) \right\rangle. \tag{43}$$

1.12. Zimm model for an ideal linear chain

The Zimm model is an extension of the Rouse model describing a so-called non-draining polymer, for which the hydroynamic interaction is also taken into account [20]. The formal approach is the same as for the Rouse model, however, the Oseen tensor or mobility matrix reads

$$\hat{\boldsymbol{H}}_{nm} = \begin{cases} \dfrac{\boldsymbol{1}}{\gamma}\delta_{nm} & n = m, \\ \dfrac{1}{8\pi\eta_s |\boldsymbol{R}_{nm}|}\left(\hat{\boldsymbol{R}}_{nm} \cdot \hat{\boldsymbol{R}}_{nm} + \boldsymbol{1}\right) & n \neq m, \end{cases} \tag{44}$$

yielding now a set of coupled differential equations

$$\frac{\partial \boldsymbol{R}_n}{\partial t} = \sum_m \hat{\boldsymbol{H}}_{nm}\left(k\frac{\partial^2 \boldsymbol{R}_n}{\partial m^2} + \boldsymbol{F}_m\right) \quad \text{with} \quad \frac{\partial}{\partial \boldsymbol{R}_m}\hat{\boldsymbol{H}}_{nm} = 0. \tag{45}$$

In order to simplify them, it is assumed that the system is sufficiently equilibrated, such that the distance-dependent parameters of the mobility matrix can be averaged over the steady state distribution. For the ideal chain under theta-solvent conditions, this distribution is Gaussian, Equation (16), and the Oseen tensor in Equation (45) is replaced by

$$\hat{\boldsymbol{H}}_{nm} \rightarrow \left\langle \hat{\boldsymbol{H}}_{nm} \right\rangle_{\mathrm{eq}} = \int d\{\boldsymbol{R}_n\}\hat{\boldsymbol{H}}_{nm}\psi_{\mathrm{eq}}(\{\boldsymbol{R}_n\}) = \frac{1}{8\pi\eta_s}\left\langle \frac{1}{\boldsymbol{R}_{nm}} \right\rangle_{\mathrm{eq}} \frac{4}{3}\boldsymbol{1} = h(n-m)\,\boldsymbol{1}$$
$$\text{for} \quad n \neq m \quad \text{with} \quad h(k) = \frac{1}{\sqrt{6\pi^3}\, k^{1/2} b\, \eta_s}. \tag{46}$$

This so-called preaveraging approximation linearizes the Langevin equation. Furthermore, using the same normal coordinates as in the Rouse model, it can be shown that the transform of $h(n-m)$ is diagonal in the mode number:

$$
\begin{aligned}
h_{pq} &= \frac{1}{N^2}\int_0^N dn \int_0^N dm \cos\left(\frac{p\pi n}{N}\right)\cos\left(\frac{q\pi m}{N}\right) h(n-m) = \frac{1}{N^2}\int_0^N dn \int_{-n}^{N-n} dk \cos\left(\frac{p\pi n}{N}\right)\cos\left(\frac{q\pi[k+n]}{N}\right) h(k) \\
&= \frac{1}{N^2}\int_0^N dn \left[\cos\left(\frac{p\pi n}{N}\right)\cos\left(\frac{q\pi n}{N}\right)\int_{-n}^{N-n} dk \cos\left(\frac{q\pi k}{N}\right) h(k) - \cos\left(\frac{p\pi n}{N}\right)\sin\left(\frac{q\pi n}{N}\right)\underbrace{\int_{-n}^{N-n} dk \sin\left(\frac{q\pi k}{N}\right) h(k)}_{=0}\right] \\
&\approx \frac{1}{N^2}\int_0^N dn \cos\left(\frac{p\pi n}{N}\right)\cos\left(\frac{q\pi n}{N}\right)\int_{-\infty}^{\infty} dk \cos\left(\frac{q\pi k}{N}\right) h(m) = \frac{1}{N^2}\int_0^N dn \cos\left(\frac{p\pi n}{N}\right)\cos\left(\frac{q\pi n}{N}\right)\frac{\sqrt{N}}{\sqrt{3\pi^3 q \eta_s b}} \\
&\approx \frac{\delta_{pq}}{\sqrt{12\pi^3 pNb^2}\,\eta_s} \approx \frac{0.0518}{\eta_s N^{1/2} b}\frac{1}{p^{1/2}}\delta_{pq} \quad \text{for} \quad p,q \neq 0, \\
h_{00} &= \frac{1}{N^2}\int_0^N dn \int_0^N dm\, h(n-m) = \frac{8}{\sqrt{54\pi^3 Nb^2}\,\eta_s} \approx \frac{0.196}{\eta_s N^{1/2} b}.
\end{aligned}
\tag{47}
$$

Thus, the differential equations for the normal coordinates are the same uncoupled ones as in the Rouse model with

$$
\gamma_p = \frac{1}{h_{pp}} \quad \text{for} \quad p = 0,1,2,\dots\ . \tag{48}
$$

For the diffusion coefficient and the relaxation times and amplitudes, we obtain

$$
\begin{aligned}
&D = \frac{k_B T}{\gamma_0} \approx 0.0798\frac{k_B T}{\eta_s R_g}, \\
&\tau_1 \approx 0.398\frac{\eta_s}{k_B T}\left(Nb^2\right)^{3/2} \approx 5.849\frac{\eta_s R_g^3}{k_B T}, \quad \tau_p = \frac{\tau_1}{p^{3/2}}, \\
&\left\langle \boldsymbol{X}_p^2 \right\rangle = \frac{3k_B T}{k_p} = \frac{Nb^2}{2\pi^2 p^2} \approx 0.0507\frac{Nb^2}{p^2} \approx 0.304\frac{R_g^2}{p^2}.
\end{aligned}
\tag{49}
$$

It is worth noting that the relaxation times are 2-fold larger than in the original publication [20], but in good agreement with more recent, corrected values [8, 21, 22] after employing the correction factor resulting from perturbation calculations [1]. Using Equation (37) one can show that the overall amplitude of segment fluctuations equals the radius of gyration:

$$
\left\langle \boldsymbol{R}'^2_{\text{seg}}(0) \right\rangle = 2\sum_{p=1}^{\infty}\left\langle \boldsymbol{X}_p^2(0) \right\rangle = R_g^2\,, \tag{50}
$$

confirming the more qualitative estimation, Equation (9), made before.

1.13. Zimm model for a swollen linear chain

For a swollen chain under good-solvent conditions, the excluded volume interaction must be taken into account. Instead of replacing the Oseen tensor by the Rotne-Prager tensor [23-27], we employ the Oseen tensor but use a non-Gaussian distance distribution, Equation (17), for the preaveraging

approximation, which we consider more appropriate for a considerably flexible and soft polymer like the chromatin fiber:

$$\left\langle \hat{\boldsymbol{H}}_{nm} \right\rangle_{\mathrm{eq}} = \frac{1}{6\pi\eta_s} \left\langle \frac{1}{\boldsymbol{R}_{nm}} \right\rangle_{\mathrm{eq}} \boldsymbol{1} = h(n-m)\boldsymbol{1} \quad \text{for} \quad n \neq m \quad \text{with} \quad h(k) \approx \frac{0.0680}{\eta_s k^{3/5} b}. \tag{51}$$

Again, the Langevin equations are linearized and diagonalized, and the difference to the ideal case is covered by modified parameters k_p, γ_p – the linearization approximation. In full analogy to the case of the ideal chain, the hydrodynamic interaction matrix can be derived:

$$\begin{aligned} h_{pq} &\approx \frac{0.0772}{\eta_s N^{3/5} b} \frac{1}{p^{2/5}} \delta_{pq} \quad \text{for} \quad p,q \neq 0, \\ h_{00} &\approx \frac{0.243}{\eta_s N^{3/5} b}. \end{aligned} \tag{52}$$

Now, the generalized spring constant can be determined using the equipartition theorem stating that each mode carries an energy of

$$\frac{k_p}{2} \left\langle \boldsymbol{X}_p^2 \right\rangle = \frac{3}{2} k_B T, \tag{53}$$

and using Equation S(35), integration by parts and the relation

$$\frac{\partial \boldsymbol{R}_n}{\partial n} \frac{\partial \boldsymbol{R}_n}{\partial n} = -\frac{1}{2} \frac{\partial^2}{\partial n\, \partial n} (\boldsymbol{R}_n - \boldsymbol{R}_m)^2 = -\frac{1}{2} \frac{\partial^2}{\partial n\, \partial n} |n-m|^{2\nu} b^2 = \nu(2\nu - 1)|n-m|^{2\nu-2} b^2 \tag{54}$$

the generalized spring constant reads

$$\begin{aligned} k_p &= \left(\frac{\left\langle \boldsymbol{X}_p^2 \right\rangle}{3 k_B T} \right)^{-1} = \left[-\frac{1}{6\pi^2 k_B T p^2} \int_0^N dn \int_0^N dm \sin\left(\frac{p\pi n}{N}\right) \sin\left(\frac{p\pi m}{N}\right) \frac{\partial^2}{\partial n\, \partial m} \left\langle (\boldsymbol{R}_n - \boldsymbol{R}_m)^2 \right\rangle_{\mathrm{eq}} \right]^{-1} \\ &\approx 71.1 \frac{k_B T}{N^{2\nu} b^2} p^{2\nu+1} \to 63.0 \frac{k_B T}{N^{2\nu} b^2} p^{2\nu+1}. \end{aligned} \tag{55}$$

In the last step, the numerical factor was adapted to fulfill for the overal amplitude of the segment fluctuations

$$\left\langle \boldsymbol{R}'^2_{\mathrm{seg}}(0) \right\rangle = 2 \sum_{p=1}^{\infty} \left\langle \boldsymbol{X}_p^2(0) \right\rangle = R_g^2 . \tag{56}$$

For the diffusion coefficient and the relaxation times and amplitudes, we obtain

$$D \approx 0.0288 \frac{k_B T}{\eta_s R_g},$$

$$\tau_1 \approx 0.206 \frac{\eta_s}{k_B T} \left(N^{3/5} b\right)^3 \approx 3.845 \frac{\eta_s R_g^3}{k_B T}, \quad \tau_p = \frac{\tau_1}{p^{9/5}}, \tag{57}$$

$$\left\langle X_p^2 \right\rangle \approx 0.0477 \frac{\left(N^{3/5} b\right)^2}{p^{11/5}} \approx 0.335 \frac{R_g^2}{p^{11/5}}.$$

1.14. Zimm model for a globular linear chain

For a globular chain under poor-solvent conditions, again the preaveraging approximation is employed, however, with a more compact Gaussian segment distance distribution, Equation (16), which we consider appropriate for the globular configuration of a considerably flexible and soft polymer like the chromatin fiber:

$$\left\langle \hat{\boldsymbol{H}}_{nm} \right\rangle_{\text{eq}} = \frac{1}{6\pi\eta_s} \left\langle \frac{1}{\boldsymbol{R}_{nm}} \right\rangle_{\text{eq}} \boldsymbol{1} = h(n-m)\boldsymbol{1} \quad \text{for} \quad n \neq m \quad \text{with} \quad h(k) = \frac{1}{\sqrt{6\pi^3} k^{1/3} b \eta_s}. \tag{58}$$

Again, the Langevin equations are linearized and diagonalized, and the difference to the ideal case is covered by modified parameters k_p, γ_p – the linearization approximation. In full analogy to the case of the ideal chain, the hydrodynamic interaction matrix can be derived:

$$h_{pq} \approx \frac{\Gamma(2/3)\delta_{pq}}{\sqrt{24\pi^{13/3} N^{2/3} b^2 p^{4/3}} \eta_s} \approx \frac{0.0231}{\eta_s N^{1/3} b} \frac{1}{p^{2/3}} \delta_{pq} \quad \text{for} \quad p,q \neq 0,$$

$$h_{00} = \frac{9}{\sqrt{150\pi^3 q N^{2/3} b^2} \eta_s} \approx \frac{0.132}{\eta_s N^{1/3} b}. \tag{59}$$

We could show, see Equation (55), that the generalized spring constant obeys

$$k_p \propto \frac{k_B T}{N^{2\nu} b^2} p^{2\nu+1}, \tag{60}$$

and the proportionality factor is adjusted such that for $\nu = 1/3$,

$$\left\langle \boldsymbol{R}'^2_{\text{seg}}(0) \right\rangle = 2 \sum_{p=1}^{\infty} \left\langle \boldsymbol{X}_p^2(0) \right\rangle = 6 k_B T \sum_{p=1}^{\infty} k_p^{-1} = R_g^2 \tag{61}$$

is fulfilled. This yields the diffusion coefficient and the relaxation times and amplitudes:

$$D \approx 0.0626 \frac{k_B T}{\eta_s R_g},$$

$$\tau_1 \approx 0.763 \frac{\eta_s}{k_B T} N b^3 \approx 7.151 \frac{\eta_s R_g^3}{k_B T}, \quad \tau_p = \frac{\tau_1}{p}, \tag{62}$$

$$\left\langle \boldsymbol{X}_p^2 \right\rangle \approx 0.0530 \frac{N^{2/3} b^2}{p^{5/3}} \approx 0.236 \frac{R_g^2}{p^{5/3}}.$$

1.15. Zimm model for an ideal circular chain

In order to obtain the dynamic parameters of an ideal circular chain under theta-solvent conditions, the concept of the Rouse model for circular chains and the preaveraging and linearization approximations of the Zimm model for ideal linear chains can be combined [19, 28, 29]. We assume for the segment distance distribution (for both theta- and good-solvent conditions)

$$\psi_{nm}(r)=\begin{cases}\left(\dfrac{3}{2\pi\mu b^2}\right)^{3/2}\exp\left(-\dfrac{r^2}{\mu b^2}\right) & \nu=\dfrac{1}{2},\\[2ex] \left(\dfrac{3}{2\pi\mu^{2/3}b^2}\right)^{3/2}\exp\left(-\dfrac{r^2}{\mu^{2/3}b^2}\right) & \nu=\dfrac{1}{3},\end{cases}\quad \text{with}$$
$$\mu(m,n,N)=\frac{|m-n|(N-m+1)}{N},\quad 0\le m,n\le N. \tag{63}$$

This is used for the preaveraging approximation in analogy to Equation (46)

$$h(k)=\frac{1}{\sqrt{6\pi^3}\eta_s b}\frac{\sqrt{N}}{\sqrt{|k|(N-|k|)}}. \tag{64}$$

Again, the Langevin equation is linearized and diagonalized: in full analogy to the case of the ideal chain, the hydrodynamic interaction matrix reads

$$\begin{aligned}
h_{pq}&=\frac{1}{N^2}\int_0^N dn\int_0^N dm\cos\left(\frac{2p\pi n}{N}\right)\cos\left(\frac{2q\pi m}{N}\right)h(n-m)=\frac{1}{N^2}\int_0^N dn\cos\left(\frac{2p\pi n}{N}\right)\int_{-n}^{N-n}dk\cos\left(\frac{q\pi[k+n]}{N}\right)h(k)\\
&=\frac{1}{N^2}\int_0^N dn\left[\cos\left(\frac{2p\pi n}{N}\right)\cos\left(\frac{2q\pi n}{N}\right)\underbrace{\int_{-n}^{N-n}dk\cos\left(\frac{2q\pi k}{N}\right)h(k)}_{=\sqrt{N}J_0(\pi q)\cos(\pi q)/\sqrt{6\pi}\eta_s b}-\cos\left(\frac{2p\pi n}{N}\right)\sin\left(\frac{2q\pi n}{N}\right)\underbrace{\int_{-n}^{N-n}dk\sin\left(\frac{2q\pi k}{N}\right)h(k)}_{=0}\right]\\
&=\frac{J_0(\pi q)\cos(\pi q)}{\sqrt{24\pi Nb^2}\eta_s}\delta_{pq}\approx\frac{0.035}{\eta_s N^{1/2}b}\frac{1}{q^{1/2}}\delta_{pq}\quad\text{for}\quad p,q\neq 0,\\
h_{00}&=\frac{1}{N^2}\int_0^N dn\int_0^N dm\,h(n-m)=\frac{1}{\sqrt{6\pi Nb^2}\eta_s}\approx\frac{0.230}{\eta_s N^{1/2}b}.
\end{aligned} \tag{65}$$

Including the same corrections from perturbation calculations as for the linear chain, see Equation (49), we obtain for the diffusion coefficient and the relaxation times and amplitudes

$$\begin{aligned}
D&=\frac{k_BT}{\gamma_0}=k_BT\,h_{00}\approx 0.0665\frac{k_BT}{\eta_s R_g},\\
\tau_1&\approx 0.147\frac{\eta_s}{k_BT}\left(Nb^2\right)^{3/2}\approx 6.111\frac{\eta_s R_g^3}{k_BT},\quad \tau_p=\frac{\tau_1}{p^{3/2}},\\
\left\langle \boldsymbol{X}_p^2\right\rangle&=\frac{3k_BT}{k_p}\approx 0.0127\frac{Nb^2}{p^2}\approx 0.152\frac{R_g^2}{p^2}.
\end{aligned} \tag{66}$$

Just as for the Rouse model for a circular chain, the Zimm model also fulfills

$$\left\langle \boldsymbol{R}'^2_{\text{seg}}(0) \right\rangle = 2\sum_{p=1}^{\infty} \left\langle \boldsymbol{X}_p^2(0) \right\rangle = \frac{1}{2} R_g^2 . \tag{67}$$

1.16. Zimm model for a swollen circular chain

For good-solvent conditions, we employ the approaches used for a swollen linear chain and an ideal circular chain. The segment distance distribution is described in analogy to Equation (17) and (63) as

$$\begin{aligned} \psi_{nm}(r) &\approx \frac{0.29}{\left(\mu^{3/5} b\right)^{3.28}} r^{0.28} \exp\left[-1.22 \left(\frac{r}{\mu^{3/5} b} \right)^{5/2} \right], \\ \mu(m,n,N) &= \frac{|m-n|(N-m+1)}{N}, \quad 0 \le m,n \le N. \end{aligned} \tag{68}$$

This yields after the preaveraging approximation for the diagonal hydrodynamic interaction matrix

$$\begin{aligned} h_{pq} &\approx \frac{0.0568}{\eta_s N^{3/5} b} \frac{1}{q^{2/5}} \delta_{pq} \quad \text{for} \quad p,q \ne 0, \\ h_{00} &\approx \frac{0.287}{\eta_s N^{3/5} b}. \end{aligned} \tag{69}$$

Again, the generalized spring constant and the mode amplitudes can be calculated using the equipartition theorem stating that each mode carries an energy of

$$\frac{k_p}{2} \left\langle \boldsymbol{X}_p^2 \right\rangle = \frac{3}{2} k_B T, \tag{70}$$

and with the same approach as used for the linear chain, yet replacing $|n-m|$ with $\mu(m,n,N)$ and executing some of the integrations numerically, the diffusion coefficient and the relaxation times and amplitudes read

$$\begin{aligned} D &\approx 0.0830 \frac{k_B T}{\eta_s R_g}, \\ \tau_1 &\approx 0.0603 \frac{\eta_s}{k_B T} N^{9/5} b^3 \approx 4.114 \frac{\eta_s R_g^3}{k_B T}, \quad \tau_p = \frac{\tau_1}{p^{17/20}}, \\ \left\langle \boldsymbol{X}_p^2 \right\rangle &\approx 0.0103 \frac{\left(N^{3/5} b\right)^2}{p^{9/4}} \approx 0.172 \frac{R_g^2}{p^{9/4}}. \end{aligned} \tag{71}$$

Again, Equation (67) is fulfilled.

1.17. Zimm model for branched polymers

As shown in the previous sections and concluded in previous publications [13, 20, 30-32], the dynamic properties of a polymer such as the diffusion coefficient as well as amplitudes and characteristic times of relaxation modes are mostly determined by the radius of gyration, which describes the effective volume occupied by the molecule, in only weak dependence on the topology and the compaction of

the molecule. This could also be confirmed for star-like branched polymers whose relaxation is akin to linear molecules [33].

Zimm and Kilb [13] showed that for a given number N of segments of a non-draining polymer under theta-solvent conditions, the relaxation time depends on the branching ratio as $\tau_1 \propto f^{-3/2}$. This is in good agreement with our findings: combining Equation (21) and (49) yields $\tau_1 \propto R_g^3 \propto (3f-2)^{3/2} / f^3 \to f^{-3/2}$ for larger f. Grest *et al.* [31] found for a large branching ratio f under good-solvent conditions and $\tau_1 \propto f^{-1.7}$, which is again in quite good agreement with our findings: Equation (23) and (57) yield $\tau_1 \propto R_g^3 \propto (3.59f-2.59)^{1.5} / f^{3.3} \to f^{-1.8}$ for larger f. Therefore we conjecture that Equation (49), (57), (66) and (71) apply to both linear/circular and branched star-/rosette-like polymers, and the branching ratio dependence is covered by the respective dependencies of the radii of gyration, Equation (21)–(24), for both small and large f.

1.18. Scaling considerations to distinguish semi-dilute from dilute and concentrated conditions

All considerations and results above were obtained assuming a dilute system, i.e., only a single chain molecule is regarded. However, at higher concentrations, this assumption is not valid and separate chains become entangled. The application of scaling laws to semi-dilute polymer solutions showed that all interactions between chain segments are effectively screened above a characteristic distance, the correlation length [8, 9]. If this is smaller than the characteristic length of the molecules, e.g. the mean squared end-to-end distance, each molecule can be described as a chain of topologically and dynamically independent 'blobs'. Their internal properties are identical to those of a single independent chain molecule, including hydrodynamic and excluded-volume interactions so that their dynamics can be described with the Rouse-Zimm formalism [23, 34-36]. The screening on length scales beyond the correlation length and corresonding times results in a purely Rouse-like behaviour of the chain of blobs on a significantly slower time scale. The transition from dilute to semi-dilute conditions sets in when the volume that a single independent chain would occupy becomes larger than the actually available volume or, in other words, when the actual global segment or monomer concentration c is higher than it would be inside a single independent chain, c^*. Using Equation (10) for a chain consisting of N Kuhn segments the critical concentration threshold is:

$$c^* = \frac{N}{3.03 R_g^3}. \tag{72}$$

The number of monomers per blob [8] is given as:

$$N^* = N\left(\frac{c^*}{c}\right)^{5/4}. \tag{73}$$

When assuming a nucleosome concentration of 140 µM [37, 38], a range of 35–80 nm for the chromatin persistence length and 1.1–5.5 nucleosmes/11 nm for the mass density, we obtain a range of 1050–12000 chromatin segments/µm^3, well above the threshold of 13–46 segments/µm^3 critical concentration. On the other hand, the nuclear volume fraction occupied by chromatin is 10–20% [38, 39], i.e., well below the threshold for a concentrated polymer solution [1]. Therefore, the concentration regime of chromatin in mammalian interphase nuclei is semi-dilute, and blob formation is likely to occur. In addition to this generic formation of independent domains, further physical and topological

constraints may contribute like the formation of loops, loop clusters or globules. Independent of the actual nature of the domains, the treatment of chromatin dynamics using the Rouse-Zimm formalism as carried out above is appropriate.

1.19. Adiabatic accessibilty of a fluctuating polymer for diffusive tracer molecules

The volume effectively occupied by a polymer molecule is characterized by its radius of gyration and Equation (10). The net volume of the polymer chain V_c, however, is determined by its contour length L and its diameter d. A repulsive interaction between the polymer and a tracer molecule of radius R_h will increase the volume, from which the tracer is excluded, such that the effective diameter of the polymer chain is increased by $2R_h$ [40]. The effective chain volume is thus

$$V_c = \frac{\pi}{4}\left(d + 2R_h\right)^2 L\,. \tag{74}$$

From a comparison of the effectively occupied volume, Equation (10), and the effective chain volume, Equation (74), the accessibility can be defined as

$$1 - \frac{V_c}{V} \approx 1 - \operatorname{erf}\left(\frac{V_c}{V}\right). \tag{75}$$

The dynamic properties of the polymer result in a fluctuating occupied volume. The time scale of these fluctuations is defined by the relaxation kinetics of the polymer as described above. When this is slower than the diffusional mobility of the tracer molecules, these fill the available volume, the complement to the effective chain volume, adiabatically. The diffusional accessibility of the polymer molecule is thus defined by the maximum rather than the mean occupied volume, i.e., in Equation (75) we replace V with $V + \delta V$, see Equation (11), and we get the dynamic accessibility

$$1 - \frac{V_c}{V + \delta V} \approx 1 - \operatorname{erf}\left(\frac{V_c}{V + \delta V}\right), \tag{76}$$

which is higher than the static accessibilty, Equation (75).

Fluorescence correlation spectroscopy of polymer relaxation

Photon correlation techniques, especially dynamic light scattering, are widely used approaches to study polymer dynamics [3, 21]. More recently, in a number of studies intramolecular dynamics have been investigated with FCS [41-48]. Especially the uncoupling of the center-of-mass (CM) diffusion from the higher order relaxation modes by trapping or tracking [45, 49] provided detailed insight into the internal dynamics, requiring a series representation of relaxation modes for a comprehensive understanding [45, 46, 48]. In summary, as shown for double-stranded DNA, the mean-squared displacement of polymer segments can be described as confined diffusion relative to the CM, the hydrodynamic interaction must be taken into account and molecules with a sufficiently large ratio of contour to persistence length, i.e., 'soft' polymers, show Zimm relaxation behaviour.

1.20. Diffusion in a harmonic potential as seen with FCS

The relaxation processes of a polymer result in local concentration fluctuations of polymer segments even for the case that the zero order relaxation, the center of mass diffusion, is negligible. Since every

relaxation mode is independent of the others and has an exponentially decaying position correlation function [1], it can be represented by a diffusion process in a harmonic potential, which is an Ornstein-Uhlenbeck process, the simplest example of a stationary Markov process with a Gaussian probability distribution at all times [50].

Let us assume Brownian motion in a harmonic potential

$$U(\boldsymbol{r}) = \frac{1}{2}(\boldsymbol{r} - \boldsymbol{r}_c)\hat{\boldsymbol{K}}(\boldsymbol{r} - \boldsymbol{r}_c) \tag{77}$$

centered at $\boldsymbol{r}_c$. The Langevin equation is

$$m\frac{d^2\boldsymbol{r}(t)}{dt^2} = -\nabla U(\boldsymbol{r}) - \hat{\boldsymbol{\Gamma}}\frac{d\boldsymbol{r}(t)}{dt} + \boldsymbol{F}(t) = -\hat{\boldsymbol{K}}[\boldsymbol{r}(t) - \boldsymbol{r}_c] - \hat{\boldsymbol{\Gamma}}\frac{d\boldsymbol{r}(t)}{dt} + \boldsymbol{F}(t) \tag{78}$$

where the random force generates the fluctuations. Assuming isotropy, i.e., the spring constant and friction matrix become scalar, and overcritical damping, i.e., the inertia term can be neglected, the Langevin equation simplifies to

$$\gamma\boldsymbol{v}(t) = \gamma\frac{d\boldsymbol{r}(t)}{dt} = k[\boldsymbol{r}(t) - \boldsymbol{r}_c] + \boldsymbol{F}(t). \tag{79}$$

The resulting additional flux is represented by a corresponding term in the Smoluchowski or Fokker-Planck equation

$$\frac{\partial c(\boldsymbol{r},t)}{\partial t} = \frac{k}{\gamma}\nabla[(\boldsymbol{r} - \boldsymbol{r}_c)c(\boldsymbol{r},t)] + D_0\nabla^2 c(\boldsymbol{r},t). \tag{80}$$

The solution in 3D under the assumption that the diffusing particle is at time zero at a position $\boldsymbol{r}_1$, $P_D(\boldsymbol{r}_2,\tau|\boldsymbol{r}_1,0) = \delta(\boldsymbol{r}_2 - \boldsymbol{r}_1)$, turns out to be

$$P_D(\boldsymbol{r}_2,\tau|\boldsymbol{r}_1,0) = \left(\frac{k}{2\pi D_0\gamma[1-\exp(-2k\tau/\gamma)]}\right)^{3/2}\exp\left[-\frac{k}{2D_0\gamma}\frac{|(\boldsymbol{r}_2 - \boldsymbol{r}_c) - (\boldsymbol{r}_1 - \boldsymbol{r}_c)\exp(-k\tau/\gamma)|^2}{1-\exp(-2k\tau/\gamma)}\right] \tag{81}$$

with the stationary distribution

$$W_D(\boldsymbol{r}) = \lim_{\tau\to\infty} P_D(\boldsymbol{r},\tau|\boldsymbol{r}_1,0) = \left(\frac{k}{2\pi D_0\gamma}\right)^{3/2}\exp\left[-\frac{k}{2D_0\gamma}(\boldsymbol{r}_2 - \boldsymbol{r}_c)^2\right]. \tag{82}$$

The position autocorrelation function of such a diffusing particle decays with $\exp(-\tau/\tau_{\text{relax}})$, $\tau_{\text{relax}} = \gamma/k$ as expected.

The application of this Greens function to the FCS theory and subsequently averaging over all positions relative to the center of the FCS focus results in the autocorrelation function

$$G(\tau) = \frac{1}{N}\left[\left(1 + \frac{1-\exp[-\tau/\tau_{\text{relax}}]}{\upsilon}\right)^{-1}\left(1 + \frac{1-\exp[-\tau/\tau_{\text{relax}}]}{\kappa^2\upsilon}\right)^{-1/2} - \left(1+\frac{1}{\upsilon}\right)^{-1}\left(1+\frac{1}{\kappa^2\upsilon}\right)^{-1/2}\right] \tag{83}$$

where N is the number of molecules in the focus, $\upsilon = \tau_D/\tau_{\text{relax}}$ the ratio of diffusion correlation time and relaxation time and $\kappa = z_0/w_0$ the structure parameter of the focal volume, i.e., the ratio of axial and lateral focal radii [51]. For an infinitely weak potential, the correlation function for free diffusion is recovered.

When disregarding the constant term in Equation (83), an estimation of the MSD of the segments can be extracted from the correlation function [47] by comparing it to

$$G(\tau) \approx \frac{1}{N}\left(1+\frac{2\left\langle\left[\boldsymbol{R}'_{\text{seg}}(t)-\boldsymbol{R}'_{\text{seg}}(0)\right]^2\right\rangle}{3w_0^2}\right)^{-1}\left(1+\frac{2\left\langle\left[\boldsymbol{R}'_{\text{seg}}(t)-\boldsymbol{R}'_{\text{seg}}(0)\right]^2\right\rangle}{3\kappa^2 w_0^2}\right)^{-1/2}. \quad (84)$$

1.21. Zimm relaxation modes in FCS

The polymer relaxation process can be described as a weighted sum of diffusive components, Equation (37), within the framework of the Zimm model for the different conditions and topologies. The weighting is based on the mode number dependence of the mode amplitudes $\left\langle \boldsymbol{X}_p^2 \right\rangle$. The only difference made between single chains (circular or linear) and branched molecules (stars or rosettes) is the dependence of the radius of gyration on the branching ratio. The relaxation time τ_1 from a fit of the model function to experimental data yields the radius of gyration according to Equation (49), (57), (62), (66) or (71), provided that the nuclear solvent viscosity is determined independently. With the knowlegde of the Kuhn segment length (through the persistence length), a branching ratio-dependent contour length can be determined. In the context of the 30 nm chromatin fiber, the genomic content is obtained.

1.22. Measuring the nucleoplasmic viscosity

The effective viscosity of the nucleoplasm affects chromatin segment diffusion. It is scale-dependent and varies between a small value for short distances as sensed by rotational diffusion and the value accessible with FCS when comparing apparent diffusion coefficients *in vivo* and in water on the scale of the observation volume, i.e., the focal volume of the confocal microscope [52, 53]. The corresponding intracellular apparent diffusion coefficient on different scales can be described using the concept of anomalous diffusion, which yields for the mean squared displacement

$$\left\langle\left[\boldsymbol{r}(t)-\boldsymbol{r}(0)\right]^2\right\rangle = \Gamma t^{\alpha} \quad \text{or} \quad t = \left\langle\left[\boldsymbol{r}(t)-\boldsymbol{r}(0)\right]^2\right\rangle^{-1/\alpha}\Gamma^{1/\alpha} \quad \text{for} \quad 0<\alpha<1 \quad (85)$$

parametrized by the transport coefficient Γ and the anomaly parameter α. For free diffusion e.g. in solution they become $\alpha = 1$ and $\Gamma = 6D$. The FCS autocorrelation function of fluorescent molecules inside living cells with the known model function for anomalous diffusion reads

$$G(\tau) = \frac{1}{N}\left[1-\Theta_T+\Theta_T \exp\left(\frac{\tau}{\tau_T}\right)\right]\left[1+\left(\frac{\tau}{\tau_D}\right)^{\alpha}\right]^{-1}\left[1+\frac{1}{\kappa^2}\left(\frac{\tau}{\tau_D}\right)^{\alpha}\right]^{-1} \quad (86)$$

where N is the mean number of molecules in the focal volume, Θ_T the fraction of molecules in a non-fluorescent state, τ_T their dwell time in this state, $\tau_D = w_0^2/4D$ the diffusional dwell time of the molecules having an apparent diffusion coefficient D inside the focal volume of a lateral diameter w_0

and a structure parameter κ. Fitting FCS data of fluorescent molecules acquired both inside cell nuclei and in solution with this model function yields diffusion times whose ratio determines the relative viscosity of the nucleoplasm compared to water on the length scale w_0 according to $\eta_{\mathrm{rel}}(w_0)=\tau_{D,\mathrm{cell}}/\tau_{D,\mathrm{solution}}$. Using the mean squared displacement, Equation (85), and the measured intracellular anomaly parameter, the relative viscosity can be calculated for any length scale

$$\eta_{\mathrm{rel}}(x)=\left(\frac{x}{w_0}\right)^{2/\alpha-2}\eta_{\mathrm{rel}}(w_0). \tag{87}$$

In order to determine the effective nucleoplasmic viscosity this is averaged over the whole range $0\le x\le w_0$:

$$\langle\eta_{\mathrm{rel}}\rangle=\frac{1}{w_0}\int_0^{w_0}dx\,\eta_{\mathrm{rel}}(x)=\eta_{\mathrm{rel}}(w_0)\cdot\frac{\alpha}{2-\alpha}=\frac{\tau_{D,\mathrm{cell}}}{\tau_{D,\mathrm{solution}}}\cdot\frac{\alpha}{2-\alpha}. \tag{88}$$

Photobleaching experiments of chromatin-associated proteins

In order to estimate binding properties of a nuclear protein, we have combined fluorescence recovery/redistribution after photobleaching (FRAP) and continuous fluorescence photobleaching (CP) with optimized experimental settings.

1.23. FRAP of a two-dimensional strip

We performed FRAP experiments with reduced numerical aperture und increased pinhole size such that bleaching occurs approximately through the complete depth of the cell nucleus [54]. Moreover, we bleached the fluorescence in a strip-like area that spans the whole nucleus in one direction and is relatively small, of width $2a$, compared to the nuclear elongation in the other direction that we refer to as x-axis. Under these conditions, we can consider the diffusional redistribution of fluorescent molecules as an effectively one-dimensional process. A solution of the diffusion equation or Fick's 2nd law of diffusion for the one-dimensional concentration distribution over time

$$\frac{\partial c(x,t)}{\partial t}=D\nabla^2 c(x,t) \tag{89}$$

under the postbleach start condition with the bleach depth p

$$c(x,0)=c_0-c_0 p[\Theta(x+a)-\Theta(x-a)] \quad \text{with} \quad 0\le p\le 1 \tag{90}$$

is found to be

$$c(x,t)=c_0-c_0\frac{p}{2}\left[\mathrm{erf}\left(\frac{a+x}{\sqrt{\mathrm{MSD}}}\right)-\mathrm{erf}\left(\frac{a-x}{\sqrt{\mathrm{MSD}}}\right)\right],\quad \mathrm{MSD}=4Dt+x_0^2 \tag{91}$$

which can be transformed directly into a fluorescence intensity distribution over time for the postbleach image series. An initial broadening of the distribution results from diffusion during bleaching and the approximate Gaussian bleach and detection profile.

To account for diffusion and association to/dissociation from immobilized binding sites with dissociation rate k_{off}, albeit in an uncoupled manner and in pseudo-first-order approximation, the distribution can be integrated over the bleach region, $-a \le x \le a$. Assuming a rapidly diffusive free fraction, f_{free}, a slowly diffusive fraction, f_{diff}, and a transiently bound and immobilized fraction, $1 - f_{\text{free}} - f_{\text{diff}}$, this yields the intensity signal over time

$$F(t) = F(t \to \infty)\left(f_{\text{free}} + f_{\text{diff}}\left[1 + p\sqrt{\frac{Dt}{\pi a^2}}\left(1 - \exp\left[-\frac{a^2}{Dt}\right]\right) - p\,\text{erf}\left(\sqrt{\frac{a^2}{Dt}}\right)\right] + p\left[1 - f_{\text{free}} - f_{\text{diff}}\right]\left[1 - \exp(-k_{\text{off}}t)\right]\right). \tag{92}$$

Fig. S11, S12 show how to experimentally assess the profile and how the two processes, binding and diffusion, contribute to the molecular redistribution in space and time.

1.24. Continuous photobleaching in the presence of two binding states

As an alternative to the FRAP approach, CP can be used to retrieve interaction properties, especially dissociation rates, for the binding and immobilization of nuclear proteins [55] because the choice of a diffraction-limited bleaching and observation volume in combination with sub-millisecond time resolution allows to decouple diffusion and binding. The FRAP analysis showed that the mobility behaviour can neither be described by a simple diffusion model nor by a simple reaction model. Moreover, the apparent diffusion coefficient obtained from FRAP using a combined diffusion and reaction model, Equation (92), is significantly smaller than measured with FCS. Therefore, we extend the established CP model function for a single association/dissociation step to two such steps, resulting in the modified model function for two bound fractions $f_{\text{bound},1}$, $f_{\text{bound},2}$ with small dissociation rates compared to the focal bleaching rate, i.e., $k_{\text{off},1}, k_{\text{off},2} \le \alpha$:

$$\begin{aligned} F(t) &= F(0) f_{\text{bound},1}\left(\frac{1}{1 + \alpha t/2 + \alpha^2 t^2/6} - \frac{12 k_{\text{off},1}/\alpha}{5 + 14 k_{\text{off},1}/\alpha}\frac{1}{1 + 3\alpha t/7}\right)\exp(-k_{\text{off},1}t) \\ &+ F(0) f_{\text{bound},2}\left(\frac{1}{1 + \alpha t/2 + \alpha^2 t^2/6} - \frac{12 k_{\text{off},2}/\alpha}{5 + 14 k_{\text{off},2}/\alpha}\frac{1}{1 + 3\alpha t/7}\right)\exp(-k_{\text{off},2}t) \\ &+ F(0)\left(f_{\text{bound},1}\frac{12 k_{\text{off},1}/\alpha}{5 + 14 k_{\text{off},1}/\alpha} + f_{\text{bound},2}\frac{12 k_{\text{off},2}/\alpha}{5 + 14 k_{\text{off},2}/\alpha} + f_{\text{free}}\right)\exp(-\beta t). \end{aligned} \tag{93}$$

The bleaching rate β takes global bleaching of the pool of fluorescent molecules into account. Fitting CP data allows to obtain th two dissociation rates of two binding states of a nuclear chromatin-binding protein.

1.25. Point FRAP in the presence of diffusion and two binding states

We could show previously [56] that point FRAP can be used to study fast diffusion and binding processes. As shown there, one must take diffusion during the photobleaching step into consideration in the presence of a fast diffusive fraction. We did this here and fitted the recovery data with a

combination of a coupled diffusion-reaction step and an uncoupled additional binding contribution, i.e., following the same scheme as used for CP. Details are as described in Im *et al.* [56].

1.26. Model for linker histone H1 binding

Based on the FRAP and CP observations and in agreement with other studies [57-61] we suggest the following model for linker histone H1 binding to nucleosomes:

- H1 has two binding sites for DNA,
- it binds loosely and reversibly at the nucleosome entry-exit site at binding site 1 with reate constants k_{on} and $k_{\text{off},1}$,
- the loosely associated complex of H1 and the nucleosome switches eventually and irreversibly to a tightly bound conformation by engaging binding site 2 with rate constant k_{switch},
- H1 dissociates from the tightly bound state with rate constant $k_{\text{off},2}$,
- re-association with chromatin only through the initial pathway.

The corresponding rate equations for the concentrations (and equivalently fractions) of free and bound H1 molecules read

$$\begin{aligned} \frac{\partial c_{\text{free}}}{\partial t} &= -k_{\text{on}} c_{\text{free}} + k_{\text{off},1} c_{\text{bound},1} + k_{\text{off},2} c_{\text{bound},2}, \\ \frac{\partial c_{\text{bound},1}}{\partial t} &= k_{\text{on}} c_{\text{free}} - k_{\text{off},1} c_{\text{bound},1} - k_{\text{switch}} c_{\text{bound},1}, \\ \frac{\partial c_{\text{bound},2}}{\partial t} &= k_{\text{switch}} c_{\text{bound},1} - k_{\text{off},2} c_{\text{bound},2}. \end{aligned} \tag{94}$$

In steady state equilibrium, i.e., $\partial c_i / \partial t = 0$ for all t, the dissociation rates and the fractions can be obtained from FRAP and CP, and for a complete description of the kinetics, the association and the switch rate can be derived using

$$\begin{aligned} k_{\text{on}} &= \frac{k_{\text{off},1} c_{\text{bound},1} + k_{\text{off},2} c_{\text{bound},2}}{c_{\text{free}}}, \\ k_{\text{switch}} &= \frac{k_{\text{off},2} c_{\text{bound},2}}{c_{\text{bound},1}}. \end{aligned} \tag{95}$$

Determination of eu- and heterochromatin nucleosome concentration from images

We used an intensity threshold-based segmentation of the nucleus to determine the mass fractions of eu- and heterochromatin. Fig. S7 shows how we selected the lower intensity threshold for the overall chromatin distribution. The mean intensity of the segmented area served as reference for the mean nucleosome concentration. Next, we set an additional upper threshold such that ~12.5% of the area (and thus volume) were excluded additionally. Selecting for intensities lying between the thresholds provided us with a mean intensity representing the euchromatin concentration. Finally, we used the second threshold as a lower limit for segmentation of heterochromatin such that the corresponding mean intensity represented the heterochromatin concentration. In summary, this yielded a mass fraction of 19.0±1.5% and 81.0±1.5% and a relative nucleosome concentration of 1.56±0.05 and 0.91±0.01 for hetero- and euchromatin, respectively, compared to the mean concentration.

Determination of nuclear solvent viscosity

To determine the effective nuclear solvent viscosity as sensed by the chromatin fiber we had to take into consideration that fiber relaxations take place on all length scales between 0 and a few 100 nm. Since nuclear diffusion usually shows a pronounced deviation from free diffusion, we had to compare nuclear diffusion properties with those in aqueous solution in order to properly estimate the effective nuclear viscosity as described above. Therefore, we injected FITC-labeled 150 kDa dextran molecules with a hydrodynamic radius of ~13 nm [62], i.e., comparable to the dimensions of the chromatin fiber, into nontransfected MCF7 cell nuclei and acquired FCS data. This yielded a cellular diffusion correlation time τ_D = 890±37 µs and an anomaly parameter α = 0.80±0.02. In aqueous solution, we obtained τ_D = 136±6 µs and α = 1. By means of Equation (88), this resulted in an effective viscosity $\langle \eta_{\mathrm{rel}} \rangle = 4.3 \pm 0.3$.

Supplementary Figures

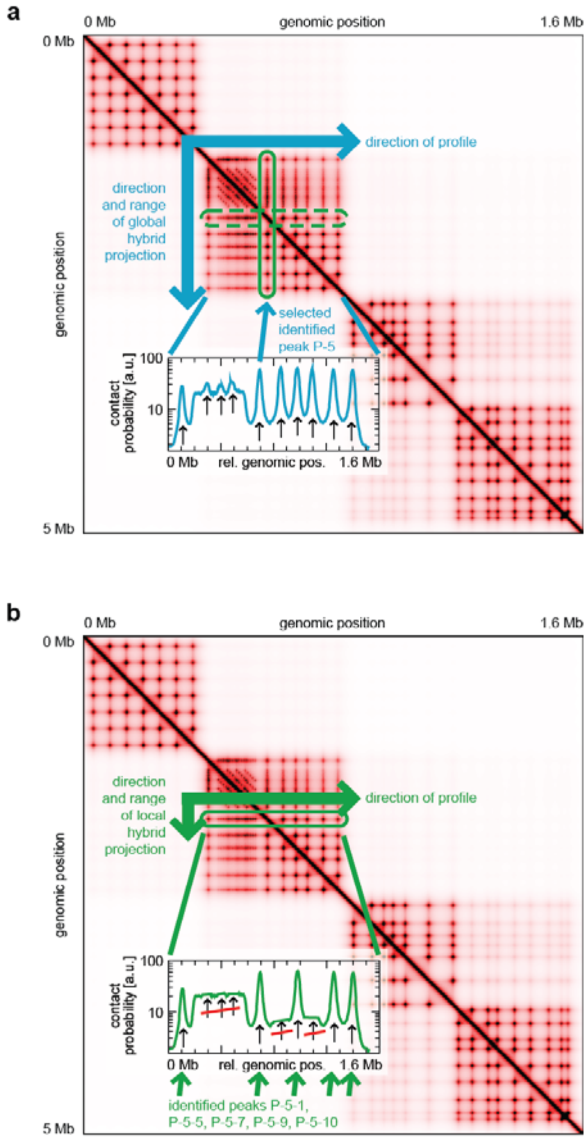

Supplementary Figure S1: Profile-based peak detection for genomic contact probability maps

a First step of peak detection, comprising a global hybrid, i.e., mean of average and maximum, projection over a manually selected domain and curve peak detection as described in Methods. **b** Second step, consisting of successive local mean of average and maximum projections at the peak locations detected in **a**, yielding pairs of interacting genomic sites.

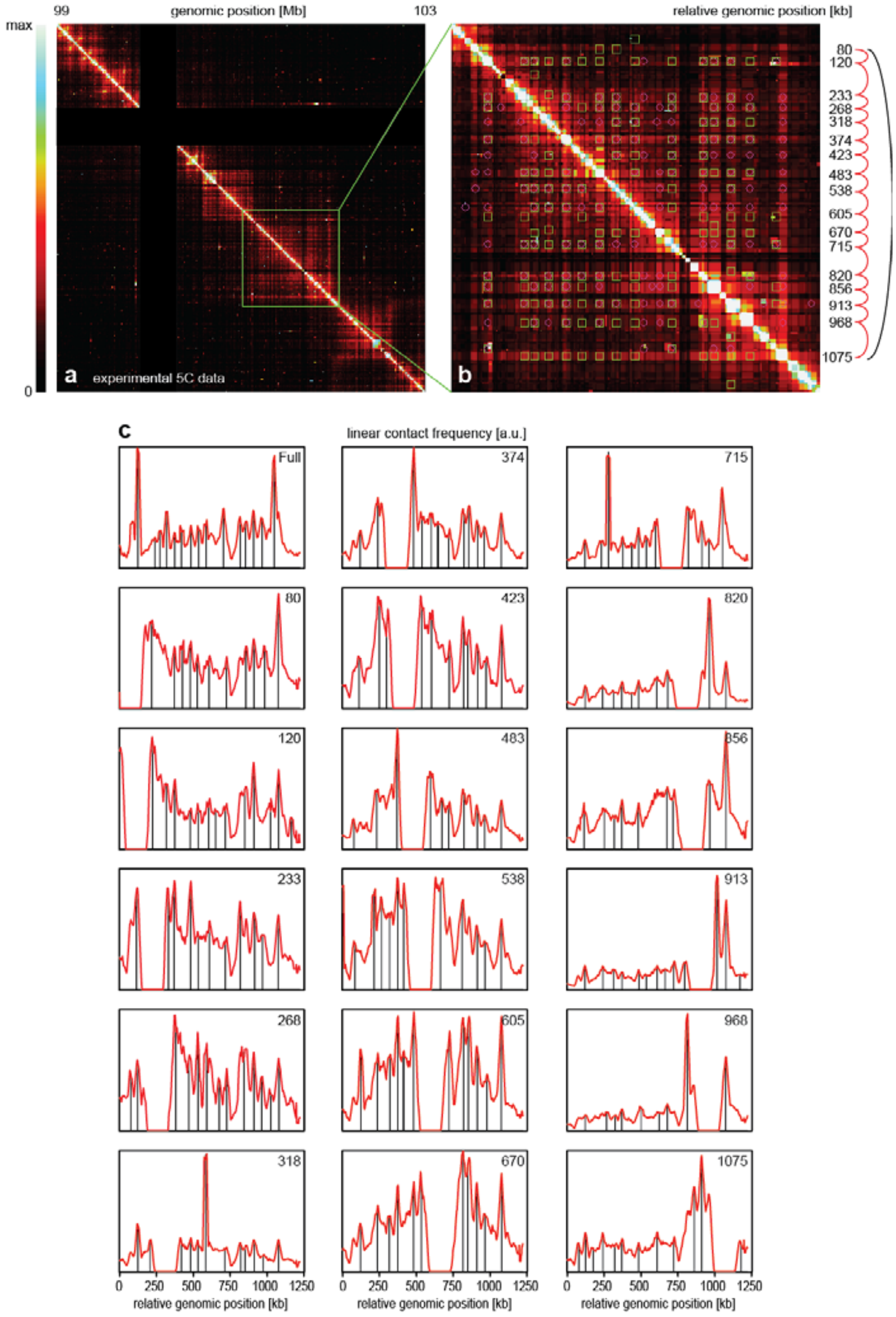

Supplementary Figure S2: Structural analysis of 5C data

a Symmetrized contact probability map extracted from experimental 5C data [63] in linear representation. The highlighted region corresponds to a domain that is shown in more detail in **b**, where the peak analysis described in Fig. S1 yielded local contact probability maxima identified in the one (green squares) and in the other projection direction (pink circles). Peaks were interpreted as sequence of loops between genomic site pairs (red arches) forming the domain (black arch). **c** Local projections (red) around the globally detected peak loci (relative genomic positions in kb given as numbers in panels) and locally detected peak loci (black).

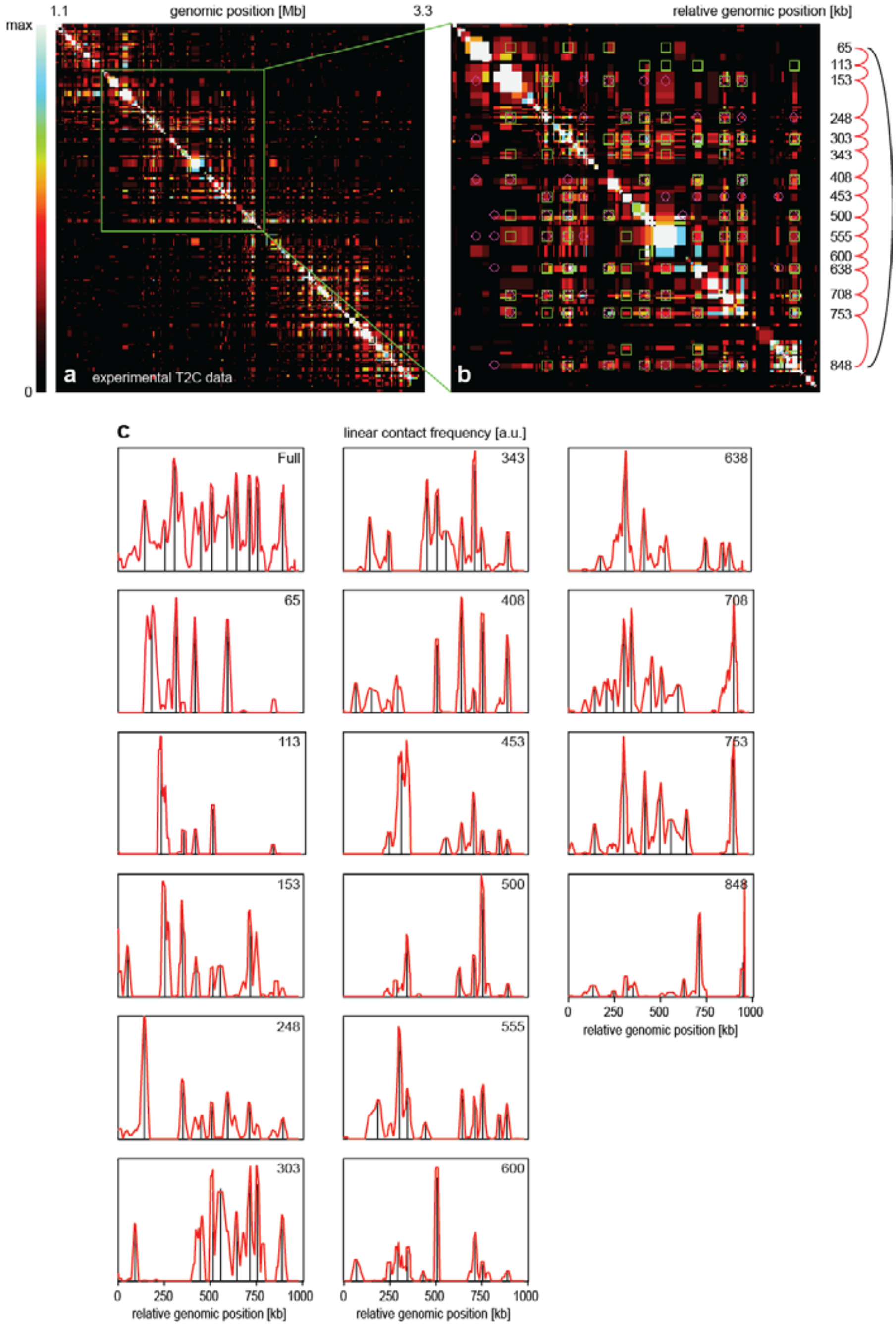

Supplementary Figure S3: Structural analysis of T2C data

a Symmetrized contact probability map extracted from experimental T2C data in linear representation. The highlighted region corresponds to a domain that is shown in more detail in **b**, where the peak analysis described in Fig. S1 yielded local contact probability maxima identified in the one (green squares) and in the other projection direction (pink circles). Peaks were interpreted as sequence of loops between genomic site pairs (red arches) forming the domain (black arch). **c** Local projections (red) around the globally detected peak loci (relative genomic positions in kb given as numbers in panels) and locally detected peak loci (black).

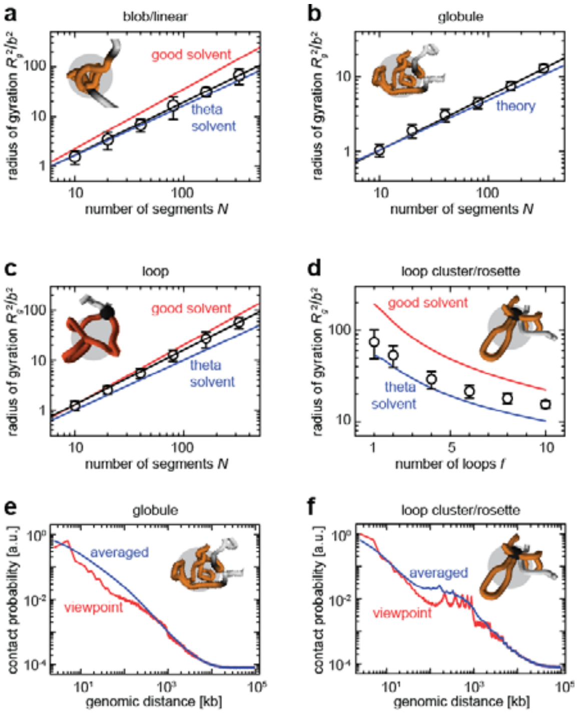

Supplementary Figure S4: Multiscale properties of simulated domain topologies

a To check the validity of the numerical model for chromatin conformations, we determined for 20 simulated conformations the averaged radius of gyration R_g for a linear chain, e.g. in a blob, depending on the number of Kuhn segments N of length b (circles). A fit with a power function (black line) yielded $R_g^2 \sim N^{1.06}$ and lay nicely between the expected dependences for theta- (blue) and good-solvent conditions (red), Equation S14. **b** Same as **a** for a globule. The fit yielded $R_g^2 \sim N^{0.73}$. **c** Same as **a** for a loop and Equation S20. The fit yielded $R_g^2 \sim N^{1.10}$. **d** Same as **a** for a loop cluster/rosette, but for N = 320 segments and depending on the number of loops per domain. The values lay nicely between the expected dependences for theta- (blue) and good-solvent conditions (red), Equation S22 and S23. **e** Linear contact probability calculated for a 20fold repetition of the globular domain configuration used for Fig. 1e and described in Methods, forming a virtual 100 Mb chromosome confined to a ~2.4 µm diameter volume. The viewpoint calculation (red) is based on a fixed first position at the beginning of the chromosome. After an initial steep decay, it shows a region with smaller slope between 100 kb and 1 Mb corresponding to the domain, followed by an again steeper decay that approaches a plateau due to the chromosomal confinement. Alternatively, the probabilities are averaged over all possible combinations (blue), resulting in a less modulated decay. **f** Same as **e** for a 20fold repetition of the loop/rosette domain configuration used for Fig. 1d and described in Methods. The viewpoint calculation (red) shows clear plateaus for the domains superimposed with peaks for the loops. The averaged calculation (blue) mostly features the first domain plateau.

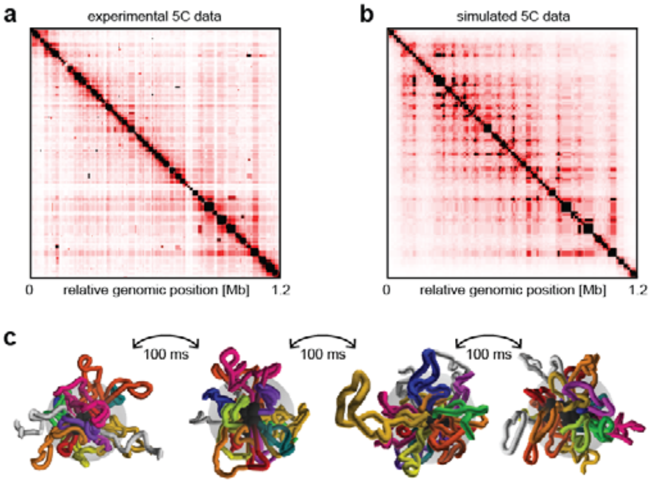

Supplementary Figure S5: Experimental and simulated 5C data and domain structure

a Experimental 5C data [63] corresponding to the highlighted domain in Fig. S2, from which peak locations where extracted. **b** Interpreting them as sequence of genomic site pairs connected by looping allowed to compute a simulated 5C map with Equation (25) at the same sampling, showing good agreement with the experimental map. **c** Renderings of statistically equivalent domain conformations formed by the loops (gray circles: radius of gyration). Transitions between such conformations are the relaxations observed with FCS and occur on the time scale of 100 ms.

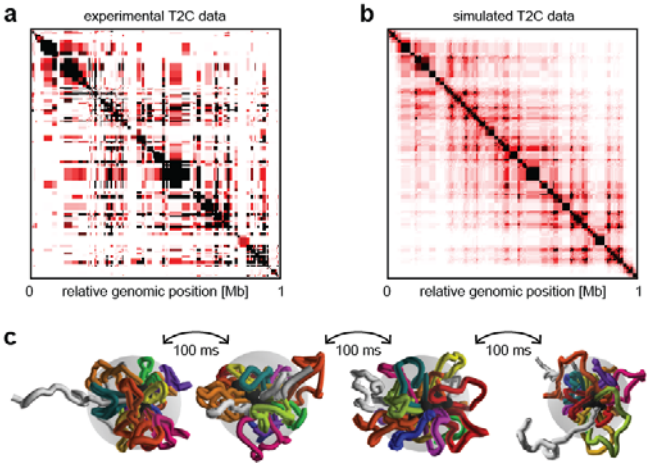

Supplementary Figure S6: Experimental and simulated T2C data and domain structure

a Experimental T2C data [64] corresponding to the highlighted domain in Fig. S3, from which peak locations where extracted. **b** Interpreting them as sequence of genomic site pairs connected by looping allowed to compute a simulated T2C map with Equation (25) at the same sampling, showing good agreement with the experimental map. **c** Renderings of statistically equivalent domain conformations formed by the loops (gray circles: radius of gyration). Transitions between such conformations are the relaxations observed with FCS and occur on the time scale of 100 ms.

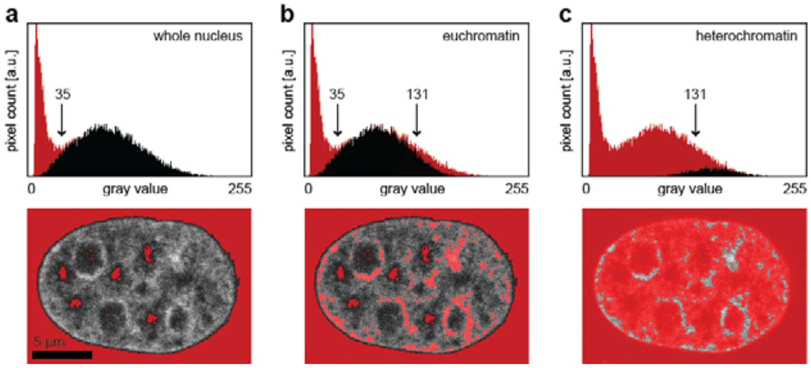

Supplementary Figure S7: Image intensity analysis and classification

a Definition of lower threshold for nuclear segmentation as minimum between background pixels (large peak left) and actual chromatin signal (smaller peak right). Disregarded pixels are highlighted in red both in the histogram and in the image. **b** The upper threshold is defined as described in the Supplementary Text, allowing to segment exclusively the euchromatin contribution. **c** The upper threshold is also used to segment exclusively the heterochromatin contribution.

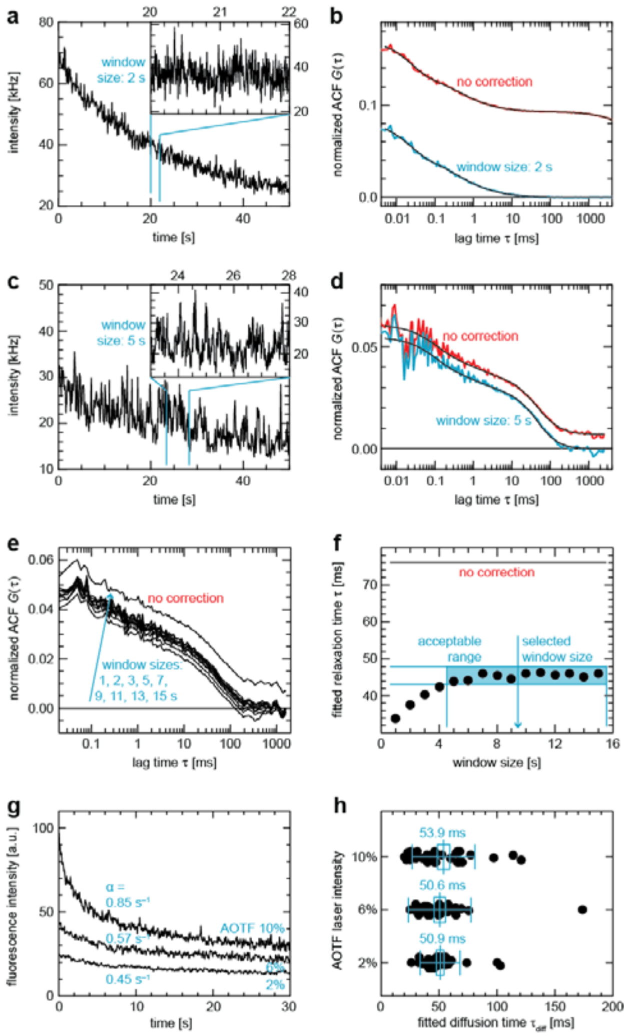

Supplementary Figure S8: FCS in the presence of photobleaching

a Fluorescence intensity trace of EYFP expressed in a HeLa cell [56], showing strong photobleaching. When zooming in to a smaller time window, the intensity is virtually stationary (insert). **b** When conventionally calculating the ACF (red), the photobleaching was reflected by an offset and a shoulder, making further analysis difficult. With a window size of 2 s, the local average-based ACF calculation, Equation S4, yielded an ACF (blue) that could be easily fitted with model functions. **c** Same as **a** for H1-EGFP in MCF7 cells as used in this study. **d** Same as **b** for a window size of 5 s. **e** Dependence of the ACF on the window size, suggesting a range of usable values. **f** Diffusion correlation times from fitting the tails of the AFCs in **e**. Without correction, the fit did not converge properly, whereas for a range of window sizes, we obtained virtually the same number so that a proper choice of the window size could be made as indicated. **g** Fluorescence intensity traces from FCS measurements of H2A-EGFP in a HeLa cell nucleus acquired with different laser intensities (2%, 6% and 10% nominal AOTF transmission), corresponding to 0.2, 0.9 and 2.2 µW excitation at 488 nm). **h** Fitting the resulting correlation functions with a pure diffusion model, Equation 5, yielded diffusion correlation times (slow component from a two-component fit) that did not depend on the laser intensity (N = 31, 31, 29 for the respective intensity values).

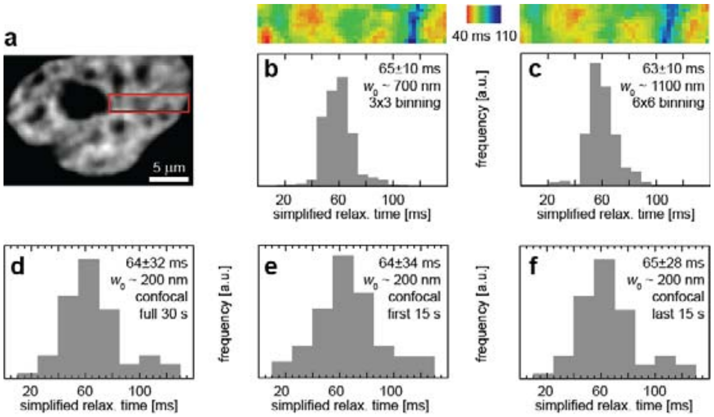

Supplementary Figure S9: Confocal and light-sheet FCS on different scales

a Light-sheet fluorescence intensity image of H2A-EGFP stably expressed in a HeLa cell (see [65] for the light-sheet imaging and 2D-FCS setup and method). Fitting the light-sheet 2D-FCS correlation functions acquired in the red region with a two-component anomalous diffusion model, Equation 5, allowed to generate maps and histograms of diffusion correlation times of the slow component. **b** 3x3 binning results in a focal volume with a radius of ~700 nm and **c** 6x6 binning in a radius of ~1100 nm. For both cases, the distributions of diffusion correlation times are very similar with almost identical mean values and standard deviations. **d** Moreover, extracting the same parameter from ~80 confocal FCS measurements (focal radius ~200 nm) in the same cell line resulted in a similar distribution and virutally the same mean value. If the relaxation times observed here were imaging artifacts, the observed relaxation times/diffusion correlation times would strongly depend on the focal size. However, they do not depend on the focal volume, strongly corroborating the interpretation of the fluctuations as polymer relaxations rather than free diffusion. **e** Analyzing only the first 30 s or **f** the second 30 s of each of the ~80 measurements from **d** yielded very similar distributions and mean values of the diffusion correlation time.

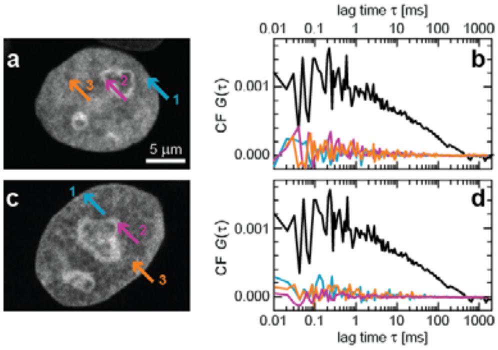

Supplementary Figure S10: FCS in fixed HeLa cells expressing H2A-EGFP

a, c Fixed HeLa cells expressing H2A-EGFP. **b, d** FCS measurements in the nuclear (1) and nucleolar (2) periphery and in the nucleoplasm (3) did not show any significant correlation, indicating that the fixation strongly suppresses the polymer relaxations as seen with FCS.

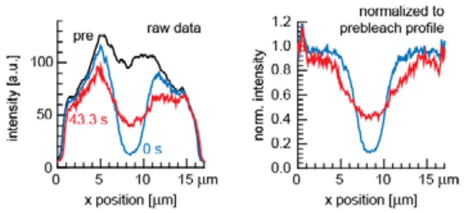

Supplementary Figure S11: Strip profile analysis of FRAP experiments

Intensity profiles extracted from the cell in Fig. 3a for the prebleach (pre), first postbleach (0 s) and a later postbleach time point (43.3 s). To remove the spatial heterogeneity distribution, the profiles were normalized to the prebleach distribution. Then they could be fitted well with Equation S91 to yield apparent diffusion coefficients.

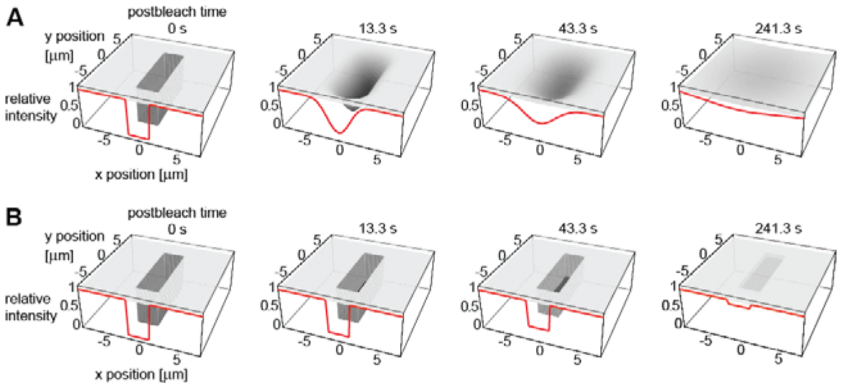

Supplementary Figure S12: Simulated FRAP experiments

a Visualized spatio-temporal distribution of purely diffusive molecules after bleaching a strip into an otherwise homogeneous distribution. The red profile represents the cross-section at $y = 0$. **b** Same as **a**, however, the molecules are immobilized such that bleached ones are only replaced following dissociation and subsequent association of still fluorescent ones. Diffusion is significantly faster than binding. Comparing the profiles revealed that (effective) diffusion contributed significantly to the H1.0 redistribution after photobleaching.

Supplementary References

1. Doi M, Edwards SF: The Theory Of Polymer Dynamics. Oxford: Oxford University Press; 1986.
2. Grosberg AY, Khokhlov AR: Statistical Physics Of Macromolecules. New York: AIP Press; 1994.
3. Berne B, Pecora R: Dynamic Light Scattering. New York: Wiley; 1976.
4. Flory PJ: Principles Of Polymer Chemistry. Ithaca, New York: Cornell University Press; 1953.
5. Kuhn W: Über die Gestalt fadenförmiger Moleküle in Lösungen. Kolloid-Z 1934,68:2-15.
6. Becker NB, Rosa A, Everaers R: The radial distribution function of worm-like chains. Eur Phys J E Soft Matter 2010,32:53-69.
7. Daniels HE: The Statistical Theory of Stiff Chains. Proc Royal Soc Edinburgh A Math 1952,63:290-311.
8. De Gennes PG: Dynamics of Entangled Polymer Solutions. II. Inclusion of Hydrodynamic Interactions. Macromolecules 1976,9:594-598.
9. Freed KF, Edwards SF: Polymer viscosity in concentrated solutions. J Chem Phys 1974,61:3626-3633.
10. Berg OG: Brownian motion of the wormlike chain and segmental diffusion of DNA. Biopolymers 1979,18:2861-2874.
11. Fujii M, Yamakawa H: Moments and Transport Coefficients of Wormlike Rings. Macromolecules 1975,8:792-799.
12. Ba X-W, Zhang S-W, Wang H-J, Wang S-J, Han Y-H: Fractal Dimension of Randomly Branched Polymers in a Good Solvent. Chin Phys Lett 2002,19:1135-1140.
13. Zimm BH, Kilb RW: Dynamics of branched polymer molecules in dilute solution. J Polymer Sci 1959,37:19-42.
14. Zimm BH, Stockmayer WH: The Dimensions of Chain Molecules Containing Branches and Rings. J Chem Phys 1949,17:1301-1314.
15. Belmont AS: Large-scale chromatin organization: the good, the surprising, and the still perplexing. Curr Opin Cell Biol 2014,26:69-78.
16. Dekker J, Rippe K, Dekker M, Kleckner N: Capturing chromosome conformation. Science 2002,295:1306-1311.
17. Gibcus JH, Dekker J: The Hierarchy of the 3D Genome. Mol Cell 2013,49:773-782.
18. Rouse PE: A Theory of the Linear Viscoelastic Properties of Dilute Solutions of Coiling Polymers. J Chem Phys 1953,21:1272-1280.
19. Bloomfield V, Zimm BH: Viscosity, Sedimentation, et Cetera, of Ring- and Straight-Chain Polymers in Dilute Solution. J Chem Phys 1966,44:315-323.
20. Zimm BH: Dynamics of Polymer Molecules in Dilute Solution: Viscoelasticity, Flow Birefringence and Dielectric Loss. J Chem Phys 1956,24:269-278.
21. Pecora R: Spectral Distribution of Light Scattered from Flexible-Coil Macromolecules. J Chem Phys 1968,49:1032-1035.
22. Sorlie SA, Pecora R: A Dynamic Light Scattering Study of a 2311 Base Pair DNA Restriction Fragment. Macromolecules 1988,21:1437-1449.
23. Bixon M: Polymer Dynamics in Solution. Annu Rev Phys Chem 1976,27:65-84.
24. Harnau L, Winkler RG, Reineker P: Dynamic structure factor of semiflexible macromolecules in dilute solution. J Chem Phys 1996,104:6355-6368.
25. Rotne J, Prager S: Variational treatment of hydrodynamic interaction in polymers. J Chem Phys 1969,50:4831-4837.
26. Yamakawa H: Transport Properties of Polymer Chains in Dilute Solution: Hydrodynamic Interaction. J Chem Phys 1970,53:436-443.
27. Zwanzig R, Kiefer J, Weiss GH: On the validity of the Kirkwood-Riseman theory. Proc Natl Acad Sci USA 1968,60:381-386.
28. Seils J, Pecora R: Photon correlation spectroscopy study of a 2311 bp relaxed circular DNA- -applicability of Rouse-Zimm and wormlike chain models. Biochem Soc Trans 1991,19:511-512.
29. Soda K: Theory of the dynamic light scattering properties for circular semiflexible chains in solution. Macromolecules 1984,17:2365-2375.
30. Allegra G, Ganazzoli F: Polymer dynamics in solution. Universal properties vs. individual behaviour in a unified approach. J Chem Soc, Faraday Trans 1997,93:2341-2353.
31. Grest GS, Kremer K, Milner ST, Witten TA: Relaxation of self-entangled many-arm star polymers. Macromolecules 1989,22:1904-1910.

32. Schaper A, Urbanke C, Maass G: Salt dependent changes in structure and dynamics of circular single stranded DNA of filamentous phages of Escherichia coli. J Biomol Struct Dyn 1991,8:1211-1232.
33. Sikorski A: Monte Carlo study of the dynamics of star-branched polymers. Macromol Theo Sim 1993,2:309-318.
34. Ahlrichs P, Everaers R, Dunweg B: Screening of hydrodynamic interactions in semidilute polymer solutions: A computer simulation study. Phys Rev E 2001,64:040501.
35. Freed KF, Perico A: Considerations on the multiple scattering representation of the concentration dependence of the viscoelastic properties of polymer systems. Macromolecules 1981,14:1290-1298.
36. Qian H: A mathematical analysis for the Brownian dynamics of a DNA tether. J Math Biol 2000,41:331-340.
37. Hihara S, Pack C-G, Kaizu K, Tani T, Hanafusa T, Nozaki T, Takemoto S, Yoshimi T, Yokota H, Imamoto N, et al: Local nucleosome dynamics facilitate chromatin accessibility in living mammalian cells. Cell Rep 2012,2:1645-1656.
38. Weidemann T, Wachsmuth M, Knoch TA, Müller G, Waldeck W, Langowski J: Counting nucleosomes in living cells with a combination of fluorescence correlation spectroscopy and confocal imaging. J Mol Biol 2003,334:229-240.
39. Knoch TA: Approaching The Three-Dimensional Organization Of The Human Genome. Ruprecht-Karls-Universität Heidelberg, Fakultät für Physik und Astronomie; 2002.
40. Minton AP: The influence of macromolecular crowding and macromolecular confinement on biochemical reactions in physiological media. J Biol Chem 2001,276:10577-10580.
41. Bonnet G, Krichevsky O, Libchaber A: Kinetics of conformational fluctuations in DNA hairpin-loops. Proc Natl Acad Sci USA 1998,95:8602-8606.
42. Bonnet G, Tyagi S, Libchaber A, Kramer FR: Thermodynamic basis of the enhanced specificity of structured DNA probes. Proc Natl Acad Sci USA 1999,96:6171-6176.
43. Edman L, Mets U, Rigler R: Conformational transitions monitored for single molecules in solution. Proc Natl Acad Sci USA 1996,93:6710-6715.
44. Lumma D, Keller S, Vilgis T, Radler JO: Dynamics of large semiflexible chains probed by fluorescence correlation spectroscopy. Phys Rev Lett 2003,90:218301.
45. McHale K, Mabuchi H: Precise Characterization of the Conformation Fluctuations of Freely Diffusing DNA: Beyond Rouse and Zimm. J Am Chem Soc 2009,131:17901-17907.
46. Petrov EP, Ohrt T, Winkler RG, Schwille P: Diffusion and Segmental Dynamics of Double-Stranded DNA. Phys Rev Lett 2006,97:258101.
47. Shusterman R, Alon S, Gavrinyov T, Krichevsky O: Monomer dynamics in double- and single-stranded DNA polymers. Phys Rev Lett 2004,92.
48. Tothova J, Brutovsky B, Lisy V: Monomer dynamics in single- and double-stranded DNA coils. Eur Phys J E Soft Matter 2007,24:61-67.
49. Cohen AE, Moerner WE: Principal-components analysis of shape fluctuations of single DNA molecules. Proc Natl Acad Sci USA 2007,104:12622-12627.
50. van Kampen NG: Stochastic Processes In Physics And Chemistry. Amsterdam: Elsevier; 1992.
51. Wachsmuth M: Fluoreszenzfluktuationsmikroskopie: Entwicklung Eines Prototyps, Theorie Und Messung Der Beweglichkeit Von Biomolekülen Im Zellkern. Ruprecht-Karls-Universität Heidelberg, Fakultät für Physik und Astronomie; 2001.
52. Baum M, Erdel F, Wachsmuth M, Rippe K: Retrieving the intracellular topology from multi-scale protein mobility mapping in living cells. Nat Comm 2014,5:4494.
53. Swaminathan R, Hoang CP, Verkman AS: Photobleaching recovery and anisotropy decay of green fluorescent protein GFP-S65T in solution and cells: cytoplasmic viscosity probed by green fluorescent protein translational and rotational diffusion. Biophys J 1997,72:1900-1907.
54. Wachsmuth M, Weisshart K: Fluorescence photobleaching and fluorescence correlation spectroscopy: Two complementary technologies to study molecular dynamics in living cells In Imaging Cellular And Molecular Biological Functions. Edited by Shorte SL, Frischknecht F: Springer; 2007: 179-228
55. Wachsmuth M, Weidemann T, Müller G, Hoffmann-Rohrer UW, Knoch TA, Waldeck W, Langowski J: Analyzing intracellular binding and diffusion with continuous fluorescence photobleaching. Biophys J 2003,84:3353-3363.
56. Im K-B, Schmidt U, Kang MS, Lee JY, Bestvater F, Wachsmuth M: Diffusion and binding analyzed with combined point FRAP and FCS. Cytometry A 2013,83:876-889.

57. Brown DT, Izard T, Misteli T: Mapping the interaction surface of linker histone H10 with the nucleosome of native chromatin in vivo. Nat Struct Mol Biol 2006,13:250-255.
58. Carrero G, Crawford E, Hendzel MJ, de Vries G: Characterizing fluorescence recovery curves for nuclear proteins undergoing binding events. Bull Math Biol 2004,66:1515-1545.
59. Catez F, Ueda T, Bustin M: Determinants of histone H1 mobility and chromatin binding in living cells. Nat Struct Mol Biol 2006,13:305-310.
60. Raghuram N, Carrero G, Stasevich TJ, McNally JG, Th'ng J, Hendzel MJ: Core histone hyperacetylation impacts cooperative behavior and high-affinity binding of histone H1 to chromatin. Biochemistry 2010,49:4420-4431.
61. Stasevich TJ, Mueller F, Brown DT, McNally JG: Dissecting the binding mechanism of the linker histone in live cells: an integrated FRAP analysis. EMBO J 2010,29:1225-1234.
62. Görisch SM, Wachsmuth M, Fejes Tóth K, Lichter P, Rippe K: Histone acetylation increases chromatin accessibility. J Cell Sci 2005,118:5825-5834.
63. Nora EP, Lajoie BR, Schulz EG, Giorgetti L, Okamoto I, Servant N, Piolot T, Berkum NLv, Meisig J, Sedat J, et al: Spatial partitioning of the regulatory landscape of the X-inactivation centre. Nature 2012,485:381-385.
64. Kolovos P, van de Werken HJ, Kepper N, Zuin J, Brouwer RW, Kockx CE, Wendt KS, van IWF, Grosveld F, Knoch TA: Targeted Chromatin Capture (T2C): a novel high resolution high throughput method to detect genomic interactions and regulatory elements. Epigenetics & chromatin 2014,7:10.
65. Capoulade J, Wachsmuth M, Knop, M: Quantitative fluorescence imaging of protein diffusion and interaction in living cells. Nat Biotechnol 2011, 29:835-839.
